# The Cord Blood Insulin and Mitochondrial DNA Content Related Methylome

**DOI:** 10.3389/fgene.2019.00325

**Published:** 2019-04-12

**Authors:** Brigitte Reimann, Bram G. Janssen, Rossella Alfano, Akram Ghantous, Almudena Espín-Pérez, Theo M. de Kok, Nelly D. Saenen, Bianca Cox, Oliver Robinson, Marc Chadeau-Hyam, Joris Penders, Zdenko Herceg, Paolo Vineis, Tim S. Nawrot, Michelle Plusquin

**Affiliations:** ^1^Centre for Environmental Sciences, University of Hasselt, Hasselt, Belgium; ^2^Epigenetics Group, International Agency for Research on Cancer (IARC), Lyon, France; ^3^Department of Biomedical Informatics Research, Stanford University, California, CA, United States; ^4^Department of Toxicogenomics, GROW School for Oncology and Developmental Biology, Maastricht University, Maastricht, Netherlands; ^5^Department of Epidemiology and Biostatistics, The School of Public Health, Imperial College London, London, United Kingdom; ^6^Medical Research Council-Health Protection Agency Centre for Environment and Health, Imperial College London, London, United Kingdom; ^7^Institute for Risk Assessment Sciences (IRAS), Division of Environmental Epidemiology, Utrecht University, Utrecht, Netherlands; ^8^Laboratory of Clinical Biology, East-Limburg Hospital, Genk, Belgium; ^9^Italian Institute for Genomic Medicine (IIGM), Turin, Italy; ^10^School of Public Health, Occupational and Environmental Medicine, KU Leuven, Leuven, Belgium

**Keywords:** insulin, mitochondrial DNA content, epigenome-wide methylation, cord blood insulin levels, mitochondrial dysfunction, differentially methylated regions, DMRs, ENVIR*ON*AGE

## Abstract

Mitochondrial dysfunction seems to play a key role in the etiology of insulin resistance. At birth, a link has already been established between mitochondrial DNA (mtDNA) content and insulin levels in cord blood. In this study, we explore shared epigenetic mechanisms of the association between mtDNA content and insulin levels, supporting the developmental origins of this link. First, the association between cord blood insulin and mtDNA content in 882 newborns of the ENVIR*ON*AGE birth cohort was assessed. Cord blood mtDNA content was established via qPCR, while cord blood levels of insulin were determined using electrochemiluminescence immunoassays. Then the cord blood DNA methylome and transcriptome were determined in 179 newborns, using the human 450K methylation Illumina and Agilent Whole Human Genome 8 × 60 K microarrays, respectively. Subsequently, we performed an epigenome-wide association study (EWAS) adjusted for different maternal and neonatal variables. Afterward, we focused on the 20 strongest associations based on *p*-values to assign transcriptomic correlates and allocate corresponding pathways employing the R packages ReactomePA and RDAVIDWebService. On the regional level, we examined differential methylation using the DMRcate and Bumphunter packages in R. Cord blood mtDNA content and insulin were significantly correlated (*r* = 0.074, *p* = 0.028), still showing a trend after additional adjustment for maternal and neonatal variables (*p* = 0.062). We found an overlap of 33 pathways which were in common between the association with cord blood mtDNA content and insulin levels, including pathways of neurodevelopment, histone modification, cytochromes P450 (CYP)-metabolism, and biological aging. We further identified a DMR annotated to Repulsive Guidance Molecule BMP Co-Receptor A (*RGMA*) linked to cord blood insulin as well as mtDNA content. Metabolic variation in early life represented by neonatal insulin levels and mtDNA content might reflect or accommodate alterations in neurodevelopment, histone modification, CYP-metabolism, and aging, indicating etiological origins in epigenetic programming. Variation in metabolic hormones at birth, reflected by molecular changes, might via these alterations predispose children to metabolic diseases later in life. The results of this study may provide important markers for following targeted studies.

## Introduction

According to the “Developmental Origins of Health and Disease” hypothesis, prenatal exposures can result in adverse health conditions later in life by increasing disease susceptibility through epigenetic programming (Barker, 1995). Metabolic factors including elevated insulin levels in early life are generally not regarded as harmful, especially when they are within physiological range. Nevertheless, small alterations may be disadvantageous over time and increase the risk of metabolic syndrome later in life.

In adults, mitochondrial dysfunction seems to play a key role in the etiology of insulin resistance (Petersen et al., 2003; Lowell and Shulman, 2005; Mogensen et al., 2007). Several detrimental environmental influences act through the generation of reactive oxygen species (ROS), which can, directly and indirectly, cause mitochondrial dysfunction (Meyer et al., 2013; Roubicek and Souza-Pinto, 2017). Also, direct interaction of stressors with components of the electron transport chain (ETC) can interfere with the ATP generation. As the baseline production of mitochondrial-generated ROS increases, the ETC itself and even the mitochondrial DNA (mtDNA) are damaged (Yakes and Van Houten, 1997). As the mtDNA primarily encodes for the components of the ETC-complexes, ROS-induced mtDNA damage potentially impairs the proper function of the ETC and in turn triggers further ROS production. Therefore, increased cellular ROS levels can have a self-amplifying effect, leading to energy depletion and mitochondrial dysfunction. One possible mechanism that links mitochondrial dysfunction to insulin resistance might be the alteration of energy-dependent epigenetic processes through changes in the energetic status of the cell. Previous research has already revealed associations between insulin levels and methylation of the mitochondrial transcription factor A (TFAM) (*r* = -0.49, *p* < 0.04; Sookoian et al., 2010), and between insulin resistance and mtDNA methylation (4.6-fold higher in insulin-resistant vs. insulin-sensitive subjects, *p* < 0.05; Zheng et al., 2015), which is indicative for an epigenetic link between both factors in adults.

Moreover, a surplus of glucose and accompanying high insulin levels in mothers with diabetes mellitus type 2 (DMT2) has been related to changes in DNA methylation of genes involved in pancreatic development and insulin secretion, and to an increased risk of diabetes in their adolescent and adult offspring (del Rosario et al., 2014). Although it has previously been shown that neonatal insulin levels are positively associated with cord blood mtDNA content (Vriens et al., 2018), the relationship between these two factors and the role of possible underlying epigenetic mechanisms in newborns is still unclear. It has already been proposed, that prenatal malnutrition-induced ROS may be the origin of mitochondrial dysfunction, leading to the epigenetic programming of an insulin resistant phenotype (Lee et al., 2005).

In this study, we explore shared epigenetic mechanisms between cord blood insulin levels and mtDNA content in order to clarify their underlying link. We first establish a relationship between cord blood insulin levels and mtDNA content in the ENVIRonmental influence ON early AGEing (ENVIR*ON*AGE) birth cohort. Hereafter, we investigate the associations between epigenome-wide DNA methylation and cord blood insulin as well as mtDNA content, by focusing on individual CpG sites along with differentially methylated regional clusters of correlated and genomically proximal CpGs (DMRs). We further identify common pathways between cord blood insulin levels and mtDNA content by correlating the methylome findings with the transcriptome.

## Materials and Methods

### Study Population

Our study employed 1309 eligible mother-newborn pairs with only singleton newborns participating in the ENVIR*ON*AGE birth cohort, situated in the province of Limburg, Belgium (Janssen et al., 2017). Recruitment took place between 2010 and 2016 at arrival in the delivery ward of the East-Limburg Hospital in Genk, Belgium. Maternal body mass index (BMI) was determined by dividing weight in kilograms by height in meters squared, based on data obtained during the first antenatal consultation. The conception date was assessed by combining data on the first day of the last menstrual period and the first ultrasonographic examination. After delivery, the mothers filled out a questionnaire regarding information about the in-house environment, education, occupation, health status, smoking, and lifestyle habits. The coding of maternal educational levels was carried out according to the following criteria: “low”, in the absence of any diploma; “middle”, in the presence of a high-school diploma; “high” in the presence of a college or university diploma. Maternal smoking status was categorized as “smoking during pregnancy” if the mother had smoked at any time point during pregnancy. Further, a distinction was made between mothers who had their first newborn or their second or more newborn. Newborns’ ethnicity was classified as either European, when two or more grandparents were of European origin, or as non-European when three or more grandparents were of non-European origin. Perinatal parameters such as newborns’ sex and birth date were collected by the hospital personnel after birth. Due to the availability of OMICs, mtDNA content and insulin measurements, the datasets for every research question were studied in a designated set, resulting in different numbers of participants.

Owing to the availability of mtDNA content and insulin measurements and missing data (as reported in detail in Supplementary Table S1), the analysis of associations between cord blood insulin and mtDNA content employed 882 participants. Epigenome-wide methylation status of the CpG sites was retrieved from cord blood samples in a subset of 197 newborns, enrolled between 2014 and 2015 in the framework of the EXPOsOMICS project (FP7; Vineis et al., 2017). The mother-newborn pairs recruited for this study were selected for their complete data on cord blood-insulin, mtDNA content and the applied covariates resulting in a final study sample size of 179 for the association with cord blood insulin and 176 for the association with cord blood mtDNA. Exclusion criteria and numbers due to missing data are reported in Supplementary Table S1.

The present study was conducted according to the principles outlined in the Helsinki Declaration (World Medical Association, 2013) and approved by the Ethical Committee of Hasselt University and the East-Limburg Hospital in Genk, Belgium. Written informed consent was obtained at the delivery ward from all participating mothers.

### Cord Blood Sample Collection

Cord blood samples were collected in BD Vacutainer^®^ Lithium Heparin, Plus Plastic K2EDTA Tubes (BD, Franklin Lakes, NJ, United States) immediately after delivery. The collected cord blood was centrifuged (3200 rpm for 15 min), buffy coat and plasma were instantly frozen at –80°C. DNA was extracted from white blood cells of the buffy coat using the QIAamp DNA mini kit (Qiagen, Inc., Venlo, the Netherlands) according to the manufacturer’s instructions. To retrieve RNA, samples were collected in PAXgene tubes (QIAGEN Benelux B.V., Antwerp, Belgium).

### Epigenome-Wide Methylation

DNA was extracted and processed at the Epigenetics Group, IARC. Specifically, DNA was bisulfite-converted using the Zymo EZ DNA methylation^TM^ kit (Zymo, Irvine, CA, United States). DNA was then hybridized to Illumina Infinium Human Methylation 450K BeadChip arrays (66) and scanned using the Illumina HiScanSQ system. After background subtraction using Illumina GenomeStudio raw intensity data were submitted to pre-processing, including normalization, using in-house software within the R statistical computing environment. Furthermore, quality control of samples was carried out and failed samples were excluded on the basis of Illumina’s detection *p*-value greater than 0.01 and bead count lower than 3. For probes using the Infinium II design additional background subtraction and dye bias correction were performed. Methylation levels at each CpG locus were expressed by Beta-values, as defined by the ratio of signal intensity originating from methylated CpGs over the sum of methylated and unmethylated CpGs. Finally, data were trimmed for outliers with values larger than 3 interquartile ranges below the first quartile or above the fourth quartile. CpG sites were annotated using the Bioconductor package IlluminaHumanMethylation450kanno.ilmn12.hg19 in R for the annotation of Illumina’s 450K methylation arrays.

### Mitochondrial DNA Content

The quantity and purity ratio (A260/280 and A260/230) of the extracted DNA was assessed by spectrometric analysis using the Nanodrop 1000 spectrophotometer (Isogen, Life Science, Belgium). Until further processing the extracted DNA was stored at -80°C. mtDNA content was assessed by taking the ratio of two mitochondrial gene copy numbers [mitochondrial forward primer from nucleotide 3212/reverse primer from nucleotide 3319 (*MTF3212/R3319*; Supplementary Table S2) and mitochondrial encoded NADH dehydrogenase 1 (*MT-ND1*)] to two single-copy nuclear control genes [acidic ribosomal phosphoprotein P0 (*RPLP0*) and beta-actin (*ACTB*)] using a quantitative real-time polymerase chain reaction (qPCR) assay. To ensure a uniform DNA input of 5 ng for each qPCR reaction, samples were diluted and checked using the Quant-iT^TM^ PicoGreen^®^ dsDNA Assay Kit (Life Technologies, Europe). The reaction was performed with 7.5 μL master mix, consisting of 5 μL/reaction Fast SYBR^®^ Green I dye 2 × (Applied Biosystems), forward and reverse primer (each 0.3 μL/reaction), and RNase free water (1.9 μL/reaction), which were aliquoted into the wells of a MicroAmp^®^ Fast Optical 384-Well Reaction Plate. To each well 2.5 μL from one of the diluted DNA samples were added to obtain a final volume of 10 μL per reaction. To account for inter-run variability and possible DNA contamination six inter-run calibrators (IRCs) and two no template controls (NTCs) were run together with the samples on each reaction plate. The thermal cycling conditions were as follows: (i) for the activation of the AmpliTaq Gold^®^ DNA-polymerase 20 s at 95°C, (ii) for denaturation 40 cycles of 1 s at 95°C and (iii) for annealing and extension of the PCR products 20 s at 60°C. Melting curve analyses were used at the end of each run to confirm the specificity of the reaction and absence of primer-dimers. Calculations of the cycle threshold (CT) values for the two mitochondrial genes were performed using “qBase” software (Biogazelle, Zwijnaarde, Belgium), which performs a normalization step relative to the nuclear reference genes by applying the ΔΔCT method and also taking the IRCs into account.

### Insulin Levels

Umbilical cord blood plasma and maternal plasma insulin levels (pmol/L) were measured by electrochemiluminescence immunoassay using a Modular–E170 (Roche, Basel, Switzerland) immunoanalyser.

### Gene Expression

Extraction of total RNA from cord blood was performed according to the manufacturer’s instructions using the miRNeasy mini kit (Qiagen, Venlo, Netherlands). Subsequently, DNase treatment (Qiagen, Venlo, Netherlands) was performed and RNA quantity and purity were determined by spectrophotometric means (Nanodrop 1000, Isogen, Life Science, Belgium). RNA integrity was assessed using the Agilent 2100 Bioanalyzer (Agilent Technologies, Amstelveen, Netherlands). Fluorescent cyanine-3-labeled cRNA was synthesized from 0.2 μg total RNA according to the Agilent one-color Quick-Amp labeling protocol (Agilent Technologies). After hybridization onto Agilent Whole Human Genome 8 × 60 K microarrays signal detection was performed using the Agilent DNA G2505C Microarray Scanner (Agilent Technologies). Preprocessing of raw data was carried out by Agilent Feature Extraction Software (Version 10.7.3.1, Agilent Technologies, Amstelveen, Netherlands) and in-house software within the R statistical computing environment.

### Statistics

#### Relationship Between Cord Blood Insulin Levels and mtDNA Content

To investigate the association between cord blood insulin levels and mtDNA content a multiple linear regression model was applied. The values for mtDNA content and cord blood insulin were log_10_ transformed to ensure normality of the data. Adjustment for platelet count and possible batch effects on the qPCR reaction was performed by calculating the residuals of the log_10_ transformed mtDNA content. In a second model, an additional adjustment was performed for newborns’ sex and gestational age, ethnicity, and birth weight as well as maternal smoking status, maternal age, early-pregnancy BMI and education, parity and season of conception. Additionally, the regression coefficients for both models were calculated, for the adjusted model expressed as partial Pearson correlation, using the ppcor package in R (Kim, 2015). Possible collinearity of variables was examined by calculating the variance inflation factor. To examine possible effects of maternal gestational diabetes on the outcome of the analysis we performed a sensitivity analysis by excluding cord blood samples of neonates born to mothers with the condition.

#### Methylation of Individual CpG Sites

The log_10_ transformed mtDNA content was denoised for platelet count by calculating the residuals of a model with mtDNA content as the dependent variable and platelet count as the independent variable. These residuals were subsequently used as the independent variable in the following analyses (the multiple linear mixed models). All samples used in the methylation analysis were processed in the same batch for the analysis of mtDNA content. Batch effects caused by the different chips and chip-positions in the methylation analysis were accounted for by treating them as random effects. Additionally, an adjustment for blood cell composition was applied by using the de-convolution approach proposed by Bakulski et al. (2016). The model included newborns’ sex and gestational age, ethnicity, and birthweight as well as maternal smoking status, maternal age, early-pregnancy BMI and education, parity and season of conception. To account for multiple testing, Bonferroni correction method was applied and individual CpGs sites were considered significant below the 5% Bonferroni significance level. To exclude maternal blood contamination in the collected cord blood we additionally analyzed all samples according to a previously described method (Morin et al., 2017). Briefly, methylation levels in a panel of 10 CpG sites were analyzed. If the predefined thresholds of five or more of the 10 CpG sites is exceeded, the sample is regarded as contaminated and dismissed from further analysis. The authors of the method give the Beta-value thresholds for two common normalization methods; SWAN and BMIQ. Because normalization in this study was performed employing an in-house method comparable to BMIQ, thresholds for the latter were applied.

#### Pathway Analyses

For the pathway analyses, we correlated the significant alterations in DNA methylation with gene expression data, to characterize possible functional consequences. Briefly, the input of the correlations consisted of (i) the 20 highest ranked CpG sites of one of the EWAS (cord blood insulin or mtDNA content), without regard to the direction of association (hyper-, or hypomethylation), and of (ii) the full set of transcripts (*n* = 29,164) for the same set of subjects. In the analyses with cord blood insulin and mtDNA content 171 and 168 samples, respectively, were available with corresponding transcripts. Batch effects of different hybridization dates on the gene expression values, and of chip and chip position on the Beta-values were corrected by calculating the residuals. CpG–transcript pairs with *p*-values below the Bonferroni significance level of 0.05/(20 × 29,164) = 8.6e–8 were considered significant. The corresponding EntrezGeneID numbers of the genes allocated to the significant transcript–probe IDs formed the final input for the two over-representation analyses (ORA). They were uploaded into the R Package RDAVIDWebService (version 1.20) (Fresno and Fernández, 2013) which retrieves data from the online pathway analysis tool, DAVID 6.8 (Huang et al., 2007),^1^ using an application programming interface (API). Furthermore, a second approach was followed by employing functionalities of the R package ReactomePA (Yu and He, 2016), developed for the analysis and visualization of the REACTOME pathway database. In both cases, the identification of enriched pathways was performed against the default background of all genes expressed in *Homo sapiens*. Pathways were selected with a minimum overlap of five genes with the input list and an EASE score below 0.01, and considered significant based on a Bonferroni 5% significance level. In case resulting pathways/disease-associations contained the same gene-sets only the one with the lowest *p-*value was retained.

#### Differentially Methylated Regions

We regressed the associations between insulin levels as well as mtDNA content and DMRs in cord blood. Since none of the various methods for the identification of DMRs has been widely accepted as the gold-standard, two methods, DMRcate and Bumphunter (70, 71) which work under different statistical assumptions were selected for this study. Additional information about both algorithms is provided in the Supplementary Text. The analysis of associations between cord blood insulin and DMRs as well as mtDNA content and DMRs was performed using both procedures and DMRs were considered significant if they reached a minimal false discovery rate (FDR) value (minfdr) of a constituting CpG site < 0.05 for DMRcate, or a family-wise error rate (FWER) value <0.05 for Bumphunter. To ensure comparability between the outcomes the first steps of the pre-processing, before the actual implementation of the specifying algorithm, were applied in parallel for both methods. Missing methylation Beta-values were imputed with values of the k nearest neighbors using a Euclidean metric provided by the Bioconductor package “impute”. Consequently, the retrieved values were logit transformed to the M-value scale for better compliance with the modeling assumptions (Du et al., 2010). Probes already identified as being cross-reactive, mapped to the sex chromosomes or within two nucleotides or closer to single nucleotide polymorphisms were filtered out as previously proposed (Chen et al., 2013). Batch effects due to the use of different chips were corrected using the “correctBatchEffect” function of the Bioconductor package BEclear (Akulenko et al., 2016).

For this study, the default smoothing parameters of DMRcate with bandwidth λ = 1000 bp and scaling factor *C* = 2 (kernel size = 500 bp) were applied. By derogation from the default settings, the FDR threshold of individual CpG sites in the “cpg.annotate” function was set to 1 in order to receive a CpG output which can be used in the subsequent step to identify DMRs.

Because for Bumphunter in scenarios with more than one covariate the use of the permutation approach is not recommended (71), “bootstrapping” (1000 bootstraps) was performed to assess the accuracy of sample estimates and create null distributions for the candidate regions. To be considered as overall significant, DMRs in this study had to contain at least two differentially methylated CpG sites. To study the overlapping associations between insulin and mtDNA content we investigated the intersection of DMRs with *p*-value <0.05.

## Results

### Population Characteristics

Demographic characteristics and perinatal factors of the mother-newborn pairs are reported in Table 1. In our study the vast majority of the newborns were Europeans. The mean (±SD) gestational age was 39.25 weeks and the mean birthweight was around 3.400 g. Mean maternal age was ca. 29 years and mean pregnancy BMI was 24.5 kg/m^2^. There were 54% of primiparous mothers. Most of the mothers reported not to have smoked during pregnancy and about half of them obtained a college or university degree. None of the mothers in the study had chronic diabetes but 34 of the 882 neonates were born to mothers with gestational diabetes, five of which received insulin treatment. The median (25th–75th percentile) cord blood insulin level measured in all samples (*n* = 882) was 32.64 pmol/L (18.75–51.68). When analyzed only in neonates born to mothers without gestational diabetes (*n* = 848), the median cord blood insulin level was unchanged (18.75–50.7), while for neonates born to mothers with gestational diabetes (*n* = 34) the median value was 37.11 pmol/L (25.29– 65.63). Cord blood mtDNA content in neonates from mothers with gestational diabetes was slightly higher compared to neonates born to mothers without gestational diabetes or the total study population [median 1.03 (0.75–1.45) vs. median 0.96 (0.74–1.27)]. The characteristics of the subsets of this population employed in the analysis of methylation data were comparable and are shown in Supplementary Table S3.

**Table 1:** Population characteristics and perinatal factors at sampling.

Characteristics	Study population (*n* = 882)
**Newborn**	
Girls, *n*	431 (49%)
Birthweight, g	3430 ± 454.72
European, *n*	771 (87%)
Gestational age, weeks	39.25 ± 1.43
Cord blood insulin, pmol/L	32.64 [18.75–51.68]
Cord blood mtDNA content	0.96 [0.74–1.27]
**Maternal**	
Age, years	29.44 ± 4.51
Pre-pregnancy BMI, kg/m^2^	24.5 ± 4.8
Education	
Low, *n*	111 (13%)
Middle, *n*	310 (35%)
High, *n*	461 (52%)
Smoking during pregnancy, *n*	112 (12%)
Parity	
1, *n*	473 (54%)
≥2, *n*	409 (46%)
Maternal insulin (*n* = 688), pmol/L	49.02 [26.78–135]
**Period of conception**	
January–March	234 (27%)
April–June	222 (25%)
July–September	227 (26%)
October–December	199 (23%)


*The numbers represent counts (percentages) for categorical and means ± standard deviation for continuous variables. mtDNA content is established by normalizing the mtDNA copy number (mean of MTF3212/R3319 and MT-ND1) to nuclear DNA copy number (mean of RPLP0 and ACTB). Cord blood mtDNA content and insulin levels are reported as median with 25th–75th percentile.*

### Association Between mtDNA Content and Cord Blood Insulin

**FIGURE 1:**
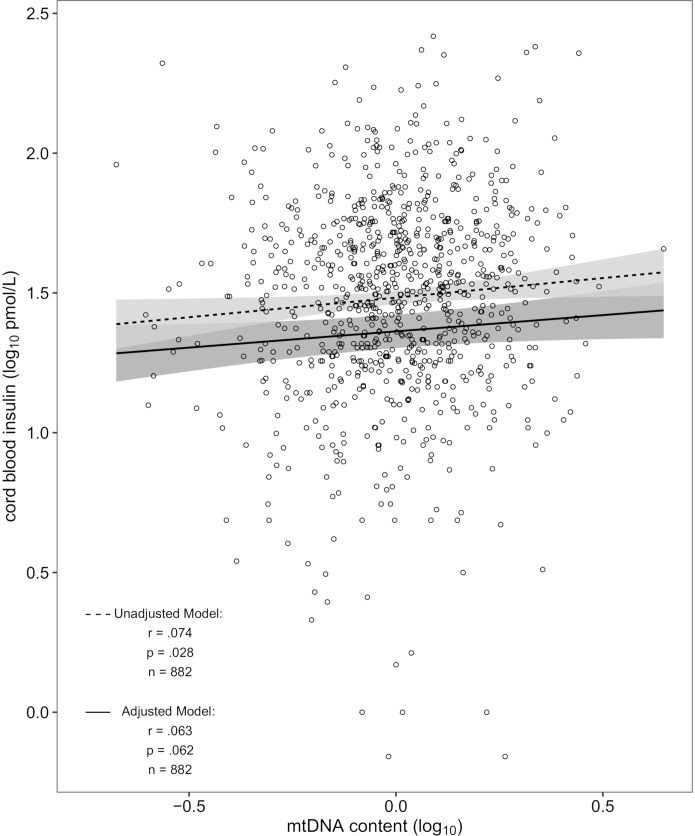
Scatterplot of cord blood mtDNA content vs. insulin in the unadjusted and adjusted model (dashed and solid regression lines respectively). The unadjusted model was denoised for platelet count and batch effect. The adjusted model was additionally corrected for newborns’ sex and gestational age, ethnicity, and birthweight as well as maternal smoking status, maternal age, early-pregnancy BMI and education, parity and season of conception.

Mitochondrial DNA content and cord blood insulin were positively correlated in the unadjusted model [*r* = 0.074, *p* = 0.028 (Figure 1)] which translates in the linear regression model into a 3.0% [95% confidence interval (CI): 0.32–5.79%] higher cord blood insulin level for an interquartile range increase in mtDNA content. After additional adjustment for newborns’ sex and gestational age, ethnicity, and birthweight as well as maternal smoking status, maternal age, early-pregnancy BMI and education, parity and season of conception, an interquartile range increase in mtDNA content was associated with a 2.50% (CI: -0.12–5.18%; *p* = 0.062) higher insulin level in cord blood. Controlling for the before mentioned covariates, the partial correlation coefficient was *r* = 0.063. In the intersect of *n* = 167 samples in common for the EWAS with cord blood insulin and mtDNA content the correlation was not significant, showing an 2% (CI: -4.68–8.90%, *p* = 0.55) increase in cord blood insulin level for an interquartile range elevation in mtDNA content for the unadjusted model and 1.87% (CI: -5.65–9.64%, *p* = 0.62) for the adjusted model. Sensitivity analysis showed no difference in effect nor significance in the unadjusted model with *n* = 882 after excluding the 34 neonates of mothers with gestational diabetes (also *r* = 0.074, *p* = 0.028). Likewise, in the adjusted model the change in effect after exclusion of the 34 cord blood samples (2.45% vs. 2.5%) and the change in significance (*p* = 0.069 vs. 0.062) were neglectable. In the same way, no notable changes were observed for the intersect of *n* = 164 [unadjusted model: 1.92% (CI: -4.77–9.07%, *p* = 0.58); adjusted model: 1.84% (CI: -5.92–9.85%, *p* = 0.64)].

### Epigenome-Wide Association Study of Cord Blood Insulin and mtDNA Content

None of the cord blood samples from the subsets of *n* = 179 and *n* = 176 showed maternal contamination according to the test panel of 10 CpG sites described above. In both subsets, 27 samples exceeded the threshold ranging from minimal two, to maximal four CpG sites, though none of them showed five or more violations of the threshold (Supplementary Figure S1). Neither the EWAS of cord blood insulin nor mtDNA content revealed any differentially methylated CpGs with a *p*-value below the Bonferroni threshold of 1.3e–7. The CpG site with the highest *ß*-coefficient and the lowest *p*-value associated with cord blood insulin (ß = 0.022; *p* = 2.15e–7) was located on the calcium voltage-gated channel auxiliary subunit alpha2delta 1 (*CACNA2D1*) gene (Supplementary Table S4). The CpG site with the lowest *p*-value associated with mtDNA (ß = 0.14; *p* = 2,88e–6) was annotated to guanine nucleotide binding protein alpha 12 (*GNA12*) (Supplementary Table S5).

### Gene Expression and Pathway Analyses

**FIGURE 2:**
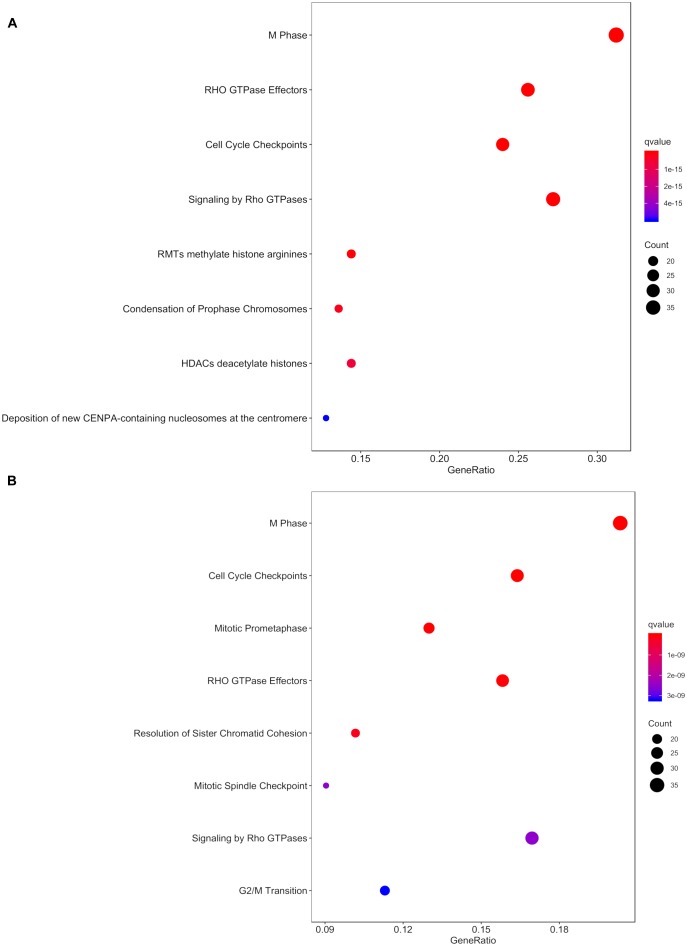
Dot plot with the q- value and count of genes from the input list contained in the resulting pathways for the association with **(A)** cord blood insulin and **(B)** mtDNA content in the analysis with ReactomePA.

**FIGURE 3:**
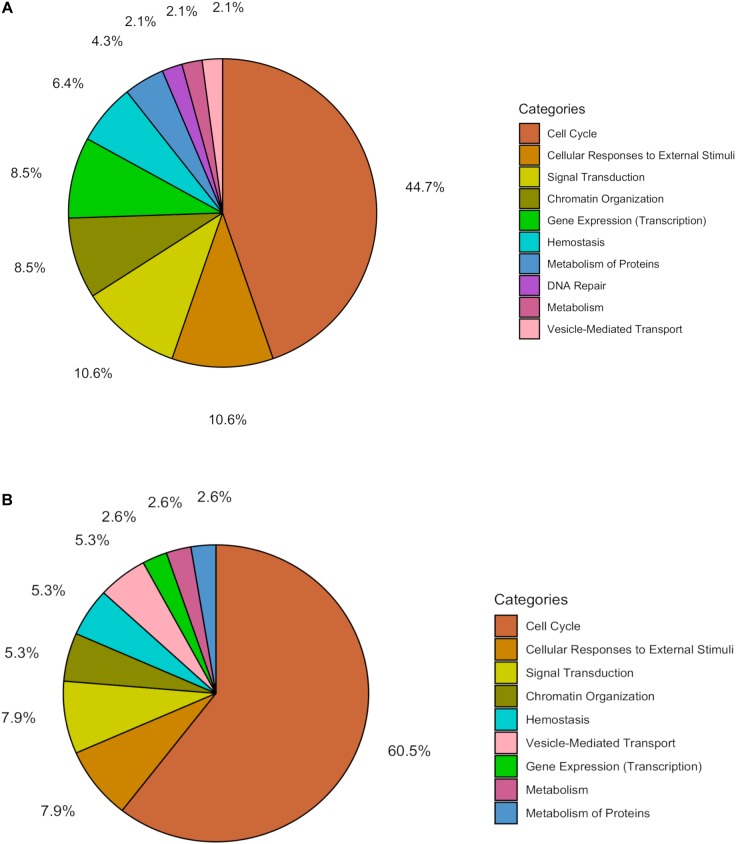
Pie chart showing the percentage of pathways for the association with **(A)** cord blood insulin and **(B)** mtDNA content within the different functional pathway categories in the analysis with ReactomePA.

Within the matrix of 20 × 29,164 = 583,280 CpG-transcript pairs we identified 233 significant pairs (*p* < 8.6e–8, Bonferroni), corresponding to 201 unique genes, associated with cord blood insulin, and 585 significant pairs (*p* < 8.6e–8, Bonferroni), corresponding to 281 unique genes, for the association with mtDNA content. The associations of the corresponding CpG-transcript pairs, and therefore the genes, with cord blood insulin and mtDNA content were in both directions, meaning, that hyper-, as well as hypomethylation, and equally transcriptional up-, as well as downregulation, were present. We found 27 enriched pathways/gene-disease-associations for cord blood insulin and 21 for mtDNA content with an overlap of 19 common pathways/gene-disease-associations in the ORA, using RDAVIDWebService (Table 2). The most significant pathway for cord blood insulin was “RMTs methylate histone arginines” and for mtDNA content “Resolution of Sister Chromatid Cohesion”. Among the pathways significant in both associations several were associated with histone modification, mitosis, and G2/M transition. Pathways associated with cord blood insulin only (Supplementary Table S6) were involved in DNA damage and repair, telomeres, and senescence. Pathways associated with mtDNA content (Supplementary Table S7) were additionally designated to “G0 and Early G1” and “Polo-like kinase-mediated events”. With the ReactomePA package, 47 and 38 pathways with a unique set of overlapping genes could be found for the correlation with cord blood insulin and mtDNA content, respectively, of which 33 were in common between both associations (Table 3). The highest ranking pathway according to the Bonferroni-corrected *p*-value in both associations was “M-Phase” (*p* = 1.61e–23 and 1.11e–14) which also showed the highest gene ratio (Figure 2). The gene ratio describes the ratio of overlap in input gene set and pathway gene set, over overlap in input gene set and background gene set. For both associations, most of the pathways fell under the Reactome category “Cell Cycle”, followed by “Cellular Responses to External Stimuli”, “Signal Transduction” and “Chromatin Organization” (Figure 3), and were associated with histone modification, neuronal development, and cellular senescence. Pathways exclusively associated with cord blood insulin or mtDNA content found with ReactomePA were comparable to those found with RDAVIDWebService (Supplementary Tables S8, S9).

**Table 2:** Pathways and gene-disease-associations commonly found for the association with cord blood insulin and mtDNA content with RDAVIDWebService.

Database	Pathway	Term	Association with cord blood insulin			Association with mtDNA content		
				
			Bonferroni-p-value (5%)	Count %	Fold enrichment	Bonferroni-p-value (5%)	Count %	Fold enrichment
REACTOME	R-HSA-3214858	RMTs methylate histone arginines	5.71E-15	8.96	18.61	1.35E-03	3.56	7.6
REACTOME	R-HSA-2299718	Condensation of Prophase Chromosomes	8.26E-14	8.46	18.29	1.01E-04	3.91	8.70
REACTOME	R-HSA-3214815	HDACs deacetylate histones	2.07E-13	8.96	15.24	9.53E-04	3.91	6.85
REACTOME	R-HSA-606279	Deposition of new CENPA-containing nucleosomes at the centromere	1.92E-12	7.96	17.21	9.48E-06	4.27	9.49
KEGG	hsa05322:	Systemic lupus erythematosus	1.19E-12	9.45	11.66	1.15E-03	4.27	5.53
REACTOME	R-HSA-2500257	Resolution of Sister Chromatid Cohesion	1.71E-11	8.96	11.84	3.88E-09	6.41	8.71
REACTOME	R-HSA-2559582	Senescence-Associated Secretory Phenotype (SASP)	5.55E-11	8.46	12.30	5.72E-04	4.27	6.39
REACTOME	R-HSA-73728	RNA Polymerase I Promoter Opening	9.90E-11	6.97	17.69	2.11E-02	2.85	7.43
REACTOME	R-HSA-2559586	DNA Damage/Telomere Stress Induced Senescence	4.00E-09	6.47	15.68	3.46E-03	3.20	7.98
REACTOME	R-HSA-68877	Mitotic Prometaphase	9.30E-09	7.46	11.06	7.77E-07	5.34	8.13
REACTOME	R-HSA-2467813	Separation of Sister Chromatids	1.70E-07	8.46	7.32	3.19E-06	6.41	5.70
REACTOME	R-HSA-5663220	RHO GTPases Activate Formins	1.92E-07	7.46	8.85	1.82E-06	5.69	6.94
REACTOME	R-HSA -189451	Heme biosynthesis	2.22E-05	2.99	43.42	1.36E-04	2.14	31.94
KEGG	hsa04110:	Cell cycle	2.11E-05	5.97	7.96	7.51E-05	4.63	6.47
KEGG	hsa04114	Oocyte meiosis	5.28E-04	4.98	7.55	7.49E-03	3.56	5.66
REACTOME	R-HSA-2565942	Regulation of PLK1 Activity at G2/M Transition	2.35E-03	4.48	8.14	5.19E-04	3.91	7.32
BIOCARTA	h_ahspPathway	Hemoglobin’s Chaperone	2.46E-03	2.49	20.83	4.34E-03	1.78	18.38
REACTOME	R-HSA-983189	Kinesins	2.29E-02	3.48	8.85	2.43E-03	3.20	8.36
REACTOME	R-HSA-69273	Cyclin A/B1/B2 associated events during G2/M transition	2.57E-02	2.49	18.09	6.63E-03	2.14	15.97


**Table 3:** Pathways and gene-disease-associations commonly found for the association with cord blood insulin and mtDNA content with ReactomePA.

Pathway	Term	Bonferroni-*p*-value (5%)	Count %	Bonferroni-*p*-value (5%)	Count %
R-HSA-176814	Activation of APC/C and APC/C:Cdc20 mediated degradation of mitotic proteins	9.27E-06	6.4	4.35E-05	5.08
R-HSA-141424	Amplification of signal from the kinetochores	4.46E-11	11.2	1.94E-08	7.91
R-HSA-176409	APC/C:Cdc20 mediated degradation of mitotic proteins	7.77E-05	5.6	2.46E-04	4.52
R-HSA-174143	APC/C-mediated degradation of cell cycle proteins	2.34E-07	8	2.75E-06	6.21
R-HSA-8854518	AURKA Activation by TPX2	6.89E-06	6.4	4.33E-06	5.65
R-HSA-69620	Cell Cycle Checkpoints	2.66E-18	24	1.29E-12	16.38
R-HSA-2559583	Cellular Senescence	1.41E-12	16	9.94E-06	8.47
R-HSA-2299718	Condensation of Prophase Chromosomes	3.35E-16	13.6	6.77E-07	6.21
R-HSA-6811434	COPI-dependent Golgi-to-ER retrograde traffic	5.34E-05	6.4	4.44E-05	5.65
R-HSA-69273	Cyclin A/B1/B2 associated events during G2/M transition	2.82E-05	4	2.20E-06	3.95
R-HSA-606279	Deposition of new CENPA-containing nucleosomes at the centromere	6.25E-15	12.8	7.18E-08	6.78
R-HSA-2559586	DNA Damage/Telomere Stress Induced Senescence	4.58E-12	11.2	9.06E-06	5.65
R-HSA-983231	Factors involved in megakaryocyte development and platelet production	7.39E-05	8	1.71E-04	6.78
R-HSA-69275	G2/M Transition	3.49E-08	12	3.21E-09	11.3
R-HSA-3214815	HDACs deacetylate histones	9.64E-16	14.4	5.30E-06	6.21
R-HSA-189451	Heme biosynthesis	5.19E-09	4.8	1.74E-07	3.39
R-HSA-983189	Kinesins	1.36E-05	5.6	4.73E-06	5.08
R-HSA-68886	M Phase	1.61E-23	31.2	1.11E-14	20.34
R-HSA-5689901	Metalloprotease DUBs	7.29E-07	5.6	2.34E-05	3.95
R-HSA-68877	Mitotic Prometaphase	2.51E-13	16.8	2.27E-11	12.99
R-HSA-68875	Mitotic Prophase	6.81E-13	14.4	4.10E-05	6.78
R-HSA-69618	Mitotic Spindle Checkpoint	3.05E-11	12	2.54E-09	9.04
R-HSA-171306	Packaging Of Telomere Ends	1.25E-10	8.8	1.78E-04	3.95
R-HSA-176408	Regulation of APC/C activators between G1/S and early anaphase	1.15E-04	5.6	3.72E-04	4.52
R-HSA-2565942	Regulation of PLK1 Activity at G2/M Transition	2.89E-06	7.2	3.22E-06	6.21
R-HSA-2500257	Resolution of Sister Chromatid Cohesion	1.15E-13	14.4	1.44E-10	10.17
R-HSA-195258	RHO GTPase Effectors	2.48E-19	25.6	3.20E-11	15.82
R-HSA-5663220	RHO GTPases Activate Formins	3.82E-10	12	3.33E-08	9.04
R-HSA-3214858	RMTs methylate histone arginines	4.77E-17	14.4	8.31E-06	5.65
R-HSA-73728	RNA Polymerase I Promoter Opening	2.51E-13	11.2	8.38E-05	4.52
R-HSA-2559582	Senescence-Associated Secretory Phenotype (SASP)	2.16E-13	13.6	3.79E-06	6.78
R-HSA-2467813	Separation of Sister Chromatids	3.73E-10	13.6	6.21E-08	10.17
R-HSA-194315	Signaling by Rho GTPases	4.37E-17	27.2	2.54E-09	16.95


### Epigenome-Wide Association Study for the Association of Differentially Methylated Regions With Cord Blood Insulin and mtDNA Content

With DMRcate we identified 20 DMRs all of which were significant on a level of FDR < 0.05 in association with cord blood insulin (Supplementary Table S10). The highest ranked DMR according to the minimal FDR value (minfdr = 4.95e–9) was located at the promoter region of the guanine nucleotide binding protein, alpha stimulating complex locus (*GNAS*) on position 57426743–57428032 of chromosome 20. The 43 CpG sites constituting this DMR were all located on a CpG island and its north shore with the majority of 36 being hypermethylated. The second highest ranking DMR (minfdr = 1.20e–7) was annotated to the Repulsive Guidance Molecule BMP Co-Receptor A (*RGMA*) gene, involved in neuronal development, overlapping its 5′ region. All 14 CpG sites were located on a CpG island and hypermethylated with the exception of two. For the association with mtDNA content, we found 34 significant DMRs with a minimal FDR < 0.05 (Supplementary Table S11 for all significant DMRs). The DMR showing the highest significance of association (minfdr = 4.53e–10) was covering an exon of the Tripartite Motif Containing 10 (*TRIM10*) gene on position 30120031–30124942 of chromosome 6. The majority of 19 of the 37 individual CpG sites constituting the DMR were located in the open sea and hypermethylated although the overall effect was a hypomethylation of the DMR. The second highest ranked DMR (minfdr = 1.16e–7) was annotated to the gene cytochrome P450 family 2 subfamily E member 1 (*CYP2E1*), overlapping the 5′ region with the individual CpG sites covering a CpG island and its south shore.

Using the Bumphunter algorithm we found no significant DMRs at a FWER < 0.05 for the association with cord blood insulin. Sixty-one DMRs had an uncorrected *p*-value < 0.05 (Supplementary Table S12) and were employed for further analysis. The highest ranking DMR (*p* = 3.5e–4) overlapped the 5′ region of the *HOXA5* gene. The CpG sites constituting this DMR were located at a CpG island and its north shore showing overall hypermethylation. In association with mtDNA content, one DMR reached significance at the FWER < 0.05 level. This DMR annotated to the gene *CYP2E1* (*p* = 6.39e–5, FWER = 0.04) and located inside an intron was significantly associated with mtDNA content. The corresponding CpG sites were located on a CpG island and its south shore, and all hypermethylated. Sixty-nine DMRs showed a *p*-value < 0.05 (Supplementary Table S13) and were subsequently applied in further investigation. In both analyses, two genes, Homeobox-A5 (*HOXA5)* and *DRD4* were represented by two different DMRs each. Both DMRs of dopamine receptor D4 (*DRD4*) were located at a CpG island, one situated at a promoter, the other at an intron, and showed hypomethylation. We found an overlap of 17 genes between the two different associations (Table 4).

**Table 4:** Overlap in annotated genes for the analysis of differentially methylated regions in the association with cord blood insulin and mtDNA content identified by the Bumphunter algorithm.

Gene	Chr	Location	Insulin						mtDNA content					
				
			Start	End	no.CpGs	Meanbetacaf	*p*-value	FWER	Start	End	no.CpGs	Meanbetacaf	*p*-value	FWER
HOXA5	chr7	promoter	27184264	27185512	17	0.014	0.0049	0.97	27183369	27183701	9	0.028	0.031917	1
HOXA5	chr7	overlaps 5′	27182493	27184030	24	0.020	0.00035	0.21	–	–	–	–	–	–
SPATC1L	chr21	overlaps 5′	47604052	47605174	8	-0.067	0.00057	0.31	47604052	47605174	8	0.052	0.018	1
ZFP57	chr6	upstream	29648161	29649084	22	0.018	0.0014	0.64	29648525	29649084	10	-0.046	0.011	1
OR2L13	chr1	overlaps 5′	248100183	248101009	11	0.044	0.0015	0.60	248100183	248101009	11	-0.052	0.0064	0.99
CYP2E1	chr10	inside intron	135341528	135343280	11	0.039	0.0021	0.72	135341528	135343280	11	0.17	6.39E-05	0.04
CPT1B	chr22	overlaps 5′	51016501	51017162	13	0.026	0.0027	0.84	51016501	51017162	13	0.037	0.0068	0.99
PIWIL1	chr12	overlaps 5′	130821607	130822818	7	0.045	0.0036	0.89	130821607	130822818	7	0.068	0.011	1.00
MUC4	chr3	upstream	195489306	195490309	9	-0.027	0.010	1.00	195488725	195490309	11	-0.051	0.0067	0.99
HLA-DQB1	chr6	promoter/overlaps 5′	32632937	32633163	8	0.030	0.011	1	32632568	32633163	11	-0.067	0.0033	0.89
RUFY1	chr5	overlaps 5′	178986131	178986906	9	-0.024	0.013	1	178986131	178986906	9	-0.077	0.0041	0.95
DRD4	chr11	inside intron	637885	639423	7	-0.031	0.014	1	637885	639423	7	-0.053	0.024	1.00
DRD4	chr11	promoter	636460	636659	2	-0.032	0.041	1	636460	636659	2	-0.102	0.011	1.00
BASP1P1	chr13	downstream	23309774	23310675	9	0.022	0.017	1	23309774	23310675	9	0.039	0.022	1
SAMD11	chr1	upstream	870791	871441	6	0.029	0.019	1	870791	871546	7	0.044	0.038	1
AURKC	chr19	overlaps 5′	57742112	57742444	9	-0.018	0.024	1	57741988	57742444	10	0.040	0.015	1
GCSAML	chr1	overlaps exon downstream	247712377	247712591	4	0.029	0.029	1	247712512	247712591	2	0.068	0.039	1
DUSP22	chr6	overlaps 5′	291882	292522	6	0.025	0.031	1	291687	293285	10	-0.038	0.016	1
HLA-E	chr6	upstream	30419491	30419612	6	-0.023	0.036	1	30420981	30421275	7	0.048	0.031	1


*Column headers: Gene = UCSC annotated gene; Location = UCSC gene region feature category; no. cpgs = number of CpGs constituting the differentially methylated region; meanbetafc = mean ß fold change; FWER = Family wise error rate. The model is adjusted for newborn’s sex and gestational age, ethnicity, and birthweight as well as maternal smoking status, maternal age, early-pregnancy BMI and education, parity and season of conception.*

When comparing the overlap of genes between the association with cord blood insulin and mtDNA content resulting from the analysis in DMRcate only the *RGMA* gene was found for both associations. A matching gene-annotation for *RGMA* was additionally found with Bumphunter for the association with mtDNA content. This DMR contained the same 12 hypermethylated CpG sites as found with DMRcate for the association with cord blood insulin. Among the 17 genes jointly found for both associations with the Bumphunter algorithm *CYP2E1* also appeared in association with mtDNA content and carnitine palmitoyltransferase 1B (*CPT1B*) in association with cord blood insulin in the results obtained with the DMRcate algorithm.

## Discussion

The origins for metabolic disorders may partly be rooted in *in utero* life (Lee et al., 2005). Here, we provide evidence for associations between cord blood insulin and mtDNA content as well as the involvement of DNA methylation as one potential underlying mechanism. Cord blood insulin levels have not been described in association with insulin resistance in later life yet, and these two parameters are therefore not interchangeable. However, evidence of predictive power of fasting insulin levels in early age with the occurrence of DMT2 later in life could also be indicative of this association in neonates. For example, a 1 SD increase in fasting insulin was associated with a relative risk of 2.04 (95% CI, 1.54–2.70) in a study investigating whether fasting insulin levels in healthy 3- to 6-year-olds are predictive of DMT2 in adulthood (Sabin et al., 2015). Since fasting insulin in children has been acknowledged as an index of insulin resistance in epidemiological studies by an expert panel (Levy-Marchal et al., 2010), it seems reasonable to also employ it as a surrogate marker in neonates.

Our observation of an association between cord blood insulin and mtDNA content in the total study population of *n* = 882 is in agreement with a previous study (Vriens et al., 2018) which also documented a positive association between cord blood insulin levels and mtDNA content. In contrast, other studies on peripheral blood found an inverse relationship between mtDNA content and insulin resistance in adolescents (Gianotti et al., 2008), in pre-diabetic subjects compared to healthy controls (Lee et al., 1998) and in offspring of DMT2 diabetic patients compared to offspring of non-diabetics (Song et al., 2001). Considering the fact, that this is the second study to find a positive association in cord blood these results may be indicative of the compensatory capacity of mitochondria, a mechanism also observed in response to other factors. We speculate that an initial increase in mtDNA content in relation to insulin during prenatal life might represent compensation for a higher metabolic demand. This is in contrast to the findings in the later course of life because the occurrence of a progressed health condition might finally mitigate the mitochondrial function resulting in lower mtDNA content. An important limitation of this study is the fact that the association between mtDNA and insulin in cord blood was not significant in the subset of *n* = 167 individuals with data on epigenome-wide methylation which may be due to mitigation of statistical power in the smaller subset. Notwithstanding, the estimated change of beta coefficients between cord blood insulin and mtDNA content was of the same order of magnitude as observed in the large sample (0.09 vs. 0.12). In addition, the omics related signature might entail a more detailed effect of the metabolic changes we observed in the larger database. Furthermore, we identified several additional mechanisms, not directly involved in mitochondrial functioning or insulin metabolism, including neuronal development, histone modification, CYP-metabolism, and biological aging, and showed associations, which have not yet been described in human neonatal studies. Several findings point in the direction of neuronal development and signaling as an important target of insulin as well as mitochondrial (dys-) function during prenatal development. We found that *CPT1B*, a regulator of the mitochondrial energy metabolism, was associated with mtDNA content and insulin. Beneficial effects on insulin sensitivity were already shown previously, in a mouse genetic model of CPT1B restriction (Kim et al., 2014). Here we found a DMR overlapping the 5′ of a CpG island and its south shore to be hypermethylated in both associations, which points in the direction of transcriptional downregulation. In somatic cells, CpG islands remain largely unmethylated, in order to allow the transcription of active genes (Jones, 2012). While there is a general consensus that methylation in the close proximity of the transcription start site (TSS) blocks transcription initiation (Jones, 2012), methylation in the gene body does not always have this effect but might rather stimulate transcription elongation, as also positive correlations between active transcription and methylation at the gene body have been reported previously (Jones, 1999; Ball et al., 2009; Yang et al., 2014; Arechederra et al., 2018). On the other hand, recent research also revealed hypermethylation of introns to be associated with transcriptional silencing (Anastasiadi et al., 2018), which further complicates the interpretation of methylation patterns in the gene body. Demethylation of specific genes, however, has been associated with an upregulation of gene expression, often in a pathological context (Wilson et al., 2007). Furthermore, we found two different DMRs annotated to *DRD4* which encodes the D4 subtype of dopamine receptors for the association with cord blood insulin as well as with mtDNA content using the Bumphunter algorithm. Activation of this receptor exerts a neuroprotective effect by inhibition of oxidative stress-induced cell death via regulation of cGMP-operated Ca^2+^ channels (Ishige et al., 2001; Shimada et al., 2010). Polymorphisms of this gene are related to various psychiatric and behavioral conditions like schizophrenia, attention deficit hyperactivity disorder and substance abuse (Ptácek et al., 2011). Associations between dopamine receptors and insulin levels have been described before (Scranton and Cincotta, 2010; Defronzo, 2011; Lamos et al., 2016), and antipsychotics, which block dopamine receptors, are linked with insulin resistance and diabetes in psychiatric patients (De Hert et al., 2011; Bobo et al., 2013). Both DMRs, containing different sets of CpG sites, were located at a CpG island, one situated at a promoter, the other at an intron, and showed hypomethylation for both associations, which could be indicative of a transcriptional upregulation. In addition, we identified DMRs annotated to *RGMA*, which has an important role in neurological development and operates through axon guidance and programmed apoptosis in the developing embryonic neural tube (Matsunaga et al., 2004; Key and Lah, 2012). *RGMA* is associated with the selective degeneration of dopaminergic neurons in the substantia nigra in a Parkinson’s disease model in mice (Korecka et al., 2017). In our study, the DMR found in association with cord blood insulin showed hypermethylation of the 5′ region at a CpG island, which is generally interpreted as transcriptional downregulation. However, the DMR found in association with mtDNA content, and located upstream in the open sea, was hypomethylated. Since *RGMA* exerts a repulsive effect on axons by stimulating the Rho-GTPase pathway (Mueller et al., 2006), the validity of this finding was reinforced by the results of the ORA, in which we identified the following three pathways in common between insulin levels and mtDNA content: “RHO GTPase Effectors”, “Signaling by Rho GTPases” and “RHO GTPases Activate Formins”. Proteins of the Rho family and their intracellular effector Rho-associated protein kinase (ROCK) engage in a range of neuronal actions especially during embryonic development with functions in axonal growth, neuronal differentiation, and dendritic spine architecture (Schmandke et al., 2007). In addition, ROCK is a key regulator of insulin by initiating cellular glucose uptake (Huang et al., 2013). Its activation was shown to cause mitochondrial dysfunction by facilitating interactions between Rac1b and cytochrome c (Kang et al., 2017).

Several pathways and DMRs found in association with cord blood insulin as well as mtDNA content showed a connection with the epigenetic mechanism of histone modification. Among these, “RMTs methylate histone arginines” and “HDACs deacetylate histones” could point in the direction of additional epigenetic modifications linking both associations. The DMR analyses revealed an association with *ZFP57*, a zinc-finger transcription factor expressed during early development and essential for the maintenance of DNA and histone methylation at imprinted control regions (Quenneville et al., 2011; Boonen et al., 2013). The output of the genome is controlled by DNA methylation, histone modification, non-coding RNAs, and chromatin remodeling which interact with each other (Alfano et al., 2018). Our finding might be interpreted as a mechanism of this cooperation.

The DMRs found at *CYP2E1* were verified in the analysis with the DMRcate package as well as with Bumphunter. *CYP2E1* encodes for a monooxygenase which metabolizes xenobiotics and is induced by ethanol, diabetic conditions, and starvation (Leung and Nieto, 2013). *CYP2E1* expression has been reported to be inhibited by insulin, and to be associated with obesity (Moncion et al., 2002; Woodcroft et al., 2002). Increased hepatic *CYP2E1* expression has also been suggested as an important cause of ROS overproduction (Aubert et al., 2011). In addition, it has been linked with mitochondrial dysfunction through oxidative damage of key components of the ETC (Bansal et al., 2010). The same DMR was found for both associations to be hypermethylated and located inside an intron on a CpG island and its south shore, which makes it difficult to infer the potential meaning in the context of transcriptional regulation.

The pathway analyses showed differential expression of genes involved in biological aging. These pathways comprised “Senescence-Associated Secretory Phenotype (SASP)” and “DNA Damage/Telomere Stress Induced Senescence” for the analysis with RDAVIDWebService, and additionally “Cellular Senescence” and “Packaging of Telomere Ends” with ReactomePA. Aging is marked by a progressive functional deterioration of cells and tissues over time (Barzilai et al., 2012; Campisi, 2013), which contribute to the etiology of metabolic diseases such as DMT2 and cardiovascular disease (Ford et al., 2002). Impaired mitochondrial quality and activity are important sources of ROS and responsible for increased damage of nuclear, mitochondrial, and telomeric DNA and therefore for cellular senescence (Passos et al., 2007). The mitochondrial energy metabolism has been associated with normal aging as well as age-related diseases (Sun et al., 2016), and also with mitochondrial dysfunction as a hallmark of organismal aging (Petersen et al., 2004). While hyperinsulinemia is a main aspect of the metabolic syndrome, conversely decreased insulin levels in diabetics are linked with accelerated aging (Bartke, 2008). Evidence from studies on caloric restriction though indicates, that suppression of circulating insulin levels decreases the risk of age-related diseases in humans (Fontana et al., 2004). The relationship between insulin levels in early life and aging in humans is therefore not clear.

We acknowledge specific strengths and limitations in the present study. We corrected the results of the multiple linear mixed models in the EWAS for the different blood-cell types, a practice that mitigates undesired effects of intra-sample heterogeneity. Furthermore, we investigated epigenome-wide DNA methylation in association with cord blood insulin and mtDNA content using an untargeted approach. This strategy allows the examination of a vast number of methylation targets in an unbiased manner. On the other hand, the large number of individual tests necessitates the rigorous correction for multiple testing with the risk of missing biologically significant associations especially in the scenario of continuous variables. We acknowledge, that the large number of tests in combination with a modest sample size reduces the power of our study. The downstream analyses of our epigenome-wide study, being overrepresentation analyses (ORA) which lead to common pathways between cord blood mtDNA content and insulin, are focused on the 20 strongest associations, based on *p*-values. The results provide important markers for following targeted studies. For the analysis on a regional level, with the DMRcate package, we chose eligible DMRs based on the minimal FDR value of constituting CpG sites instead of the Stouffer combined *p*-value. This approach was also applied in other studies (Solomon et al., 2017; Roberts et al., 2018). A limitation of the performed overrepresentation analyses (ORA) is the fact that it is not possible to retrieve the direction of the effect and therefore distinguish if the reported pathways are down-regulated. On the other hand, these limitations of the study were partially ameliorated by the multilevel exploration of methylation changes, and use of individual CpG sites as well as DMRs which provide increased biological significance to findings (Jaffe et al., 2012). Additionally, the application of two different algorithms for the identification of DMRs added verification to the obtained results. Moreover, by integrating gene-expression data and subsequent pathway analyses we were able to link epigenetic outcomes to biological consequences and thus provide mechanistic insights. A validation by future studies may enforce these hypothesis-generating findings. Although not the scope of this study, internal or external exposures such as air pollution might play a role in cord blood insulin levels (Madhloum et al., 2017) as well as in cord blood mtDNA content (Rosa et al., 2017) and the methylome (Plusquin et al., 2017; Alfano et al., 2018).

In conclusion, in our investigation of epigenome-wide methylation, we identified several previously described as well as novel genes and pathways on multiple levels in relation to both, cord blood insulin and mtDNA content. Possible important targets of insulin as well as mitochondrial (dys-) function during embryogenesis point in the direction of neuronal development, histone modification, CYP-metabolism, and biological aging.

## Ethics Statement

This study was carried out in accordance with the recommendations of “Comité voor Medische Ethiek UHasselt” and “Comité Medische Ethiek ZOL” with written informed consent from all subjects. All subjects gave written informed consent in accordance with the Declaration of Helsinki. The protocol was approved by the “Comité voor Medische Ethiek UHasselt” and “Comité Medische Ethiek ZOL”.

## Author Contributions

BR, MP, and TN designed the research hypothesis. BR, RA, and MP analyzed the data, interpreted the results, and drafted the article. JP, BJ, and NS performed the laboratory analysis of the samples with regard to cord blood insulin levels and mtDNA content. PV, AEP, TdK, AG, and ZH were responsible for the analyses of DNA methylation and the transcriptome. TN, PV, MC-H, OR, and BC provided critical revision of the manuscript with respect to the content. All authors read and approved the final manuscript.

## Conflict of Interest Statement

The authors declare that the research was conducted in the absence of any commercial or financial relationships that could be construed as a potential conflict of interest.

## Footnotes

1 https://david.ncifcrf.gov

## Abbreviations

5hmC: 5-hydroxymethylcytosine
5′UTR: 5′ untranslated region
ARHGEF10L: Rho guanine nucleotide exchange factor 10-like protein
ATP: adenosine triphosphate
BMI: body mass index
CACNA2D1: calcium voltage-gated channel auxiliary subunit alpha2delta 1
cGMP: cyclic guanosine monophosphate
CPT1B: carnitine palmitoyltransferase 1B
CYP: cytochromes P450
CYP2E1: cytochrome P450 family 2 subfamily E member 1
DMRs: differentially methylated regions
DRD4: dopamine receptor D4
EASE: expression analysis systematic explorer
ENVIR*ON*AGE: ENVIRonmental influence *ON* AGEing in early life
ETC: electron transport chain
EWAS: epigenome-wide association studies
FDR: false discovery rate
FWER: family-wise error rate
GNA12: guanine nucleotide binding protein alpha 12
HDAC: histone deacetylase
HOXA5: homeobox A5
meanbetafc: mean ß fold change
MHC: human major histocompatibility complex
mtDNA: mitochondrial DNA
nDNA: nuclear DNA
no.cpgs: number of CpGs constituting the differentially methylated region
NTCs: no template controls
qPCR: quantitative (realtime) polymerase chain reaction
RGMa: repulsive guidance molecule a
ROCK: Rho-associated protein kinase
ROS: reactive oxygen species
SASP: senescence-associated secretory phenotype
T2DM: type 2 diabetes
TFAM: mitochondrial transcription factor A
TNDM1: transient neonatal diabetes mellitus type 1
TSS: transcription start site
ZFP57: zinc finger protein 57 homolog
