# Supplementary material for: The Cord Blood Insulin and Mitochondrial DNA Content Related Methylome
Source: Front Genet. 2019 Apr 12;10:325. doi: 10.3389/fgene.2019.00325 (PMC6474284; doi:10.3389/fgene.2019.00325)
Supplement: Supplementary file 1 [file Data_Sheet_1.DOCX]

Supplementary Material

**Contents**

[**1 Supplementary Figures and Tables** 1](#_Toc531242349)

[**1.1 Supplementary Figures**  1](#_Toc531242349)

[**Figure S1** Test for cord blood contamination 1](#_Toc531242350)

[**1.2 Supplementary Tables**  1](#_Toc531242349)

[**Table S1.** Overview sample sizes 1](#_Toc531242350)

[**Table S2.** Primer sequences 2](#_Toc531242351)

[**Table S3.** Population characteristics 2](#_Toc531242352)

[**Table S4.** CpGs EWAS cord blood insulin 2](#_Toc531242353)

[**Table S5.** CpGs EWAS mtDNA content 3](#_Toc531242354)

[**Table S6.** Pathways for cord blood insulin with RDAVIDWebServices 4](#_Toc531242355)

[**Table S7.** Pathways for mtDNA content with RDAVIDWebServices 4](#_Toc531242356)

[**Table S8.** Pathways for cord blood insuilin with ReactomePA 4](#_Toc531242358)

[**Table S9.** Pathways for mtDNA with ReactomePA 5](#_Toc531242359)

[**Table S10.** Results DMRcate for cord blood insulin 5](#_Toc531242360)

[**Table S11.** Results DMRcate for mtDNA content 6](#_Toc531242361)

[**Table S12**. Results Bumphunter for cord blood insulin 7](#_Toc531242362)

[**Table S13**. Results Bumphunter for mtDNA content 8](#_Toc531242363)

**2** [**Supplementary text** Differences in algorithm between DMRcate and Bumphunter 1](#_Toc531242364)**0**

# Supplementary Figures and Tables

## Supplementary Figures


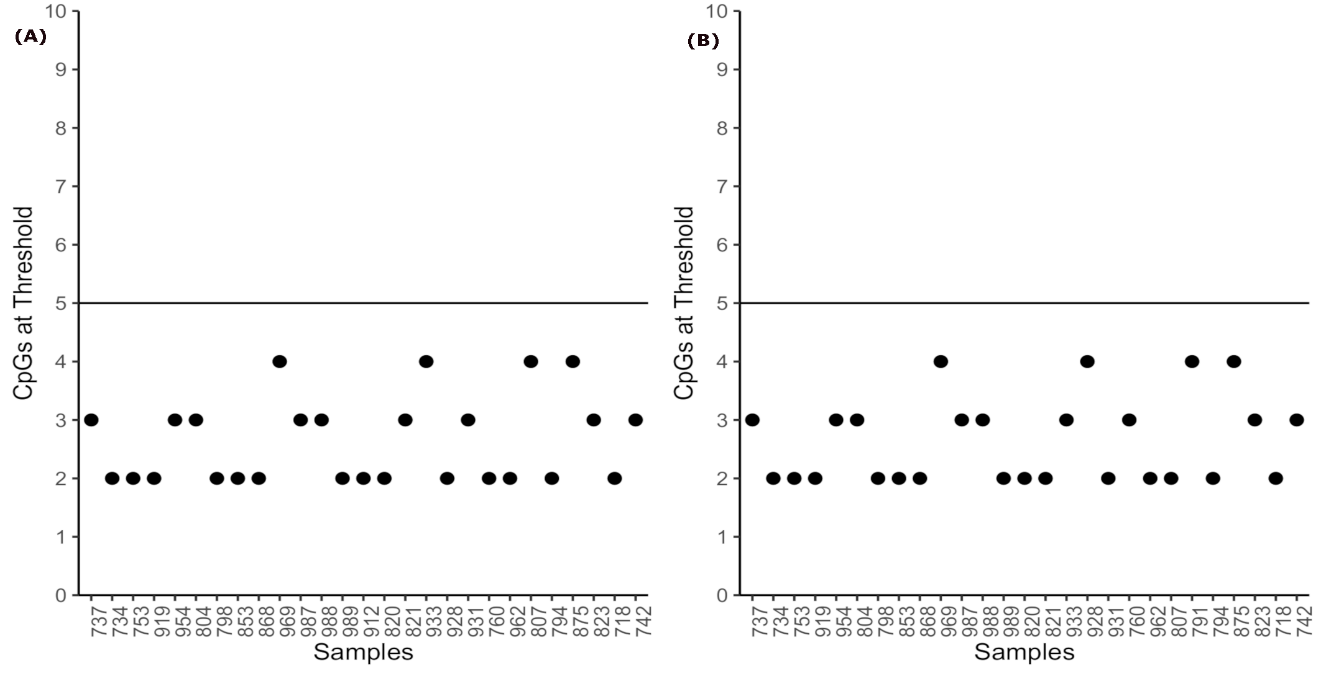


**Supplementary Figure S1 |** DNA methylation at 10 autosomal CpGs was tested to identify samples contaminated with maternal blood. Count on the number of sites over thresholds in all samples with at least one site over the threshold (x-axis) in (A) the subset of n = 179 samples for the EWAS with cord blood insulin, and (B) in the subset of n = 176 samples for the EWAS with cord blood mtDNA content. Contamination was declared if at least 5 of the 10 CpGs were above the threshold.

## Supplementary Tables

**Supplementary Table S1 |** Overview on exclusion of samples due to missing data for the analysis of correlation between cord blood insulin and mtDNA content and epigenome-wide methylation.

| **Exclusion Criterion** |  | **Number of mother-newborn pairs in the data sets** | |  |  |
| --- | --- | --- | --- | --- | --- |
|  | **Correlation insulin/ mtDNA content** | | **EWAS Insulin** | **EWAS mtDNA content** | **Intersect** |
| **Initial number** | 1309 | | 197 | 197 | 197 |
| mtDNA content | 1154 | | - | 191 | - |
| Platelet count | 961 | | - | 186 | - |
| Cord blood insulin | 911 | | 190 | - | - |
| Maternal education | 886 | | 179 | 176 | - |
| Maternal smoking | 882 | | 179 | 176 | - |
| **Final number** | 882 | | 179 | 176 | 167 |

**Supplementary** **Table S2 |** Primer sequences for mitochondrial and nuclear genes. Abbreviations: ACTB, beta actin; MTF3212/R3319, mitochondrial forward primer from nucleotide 3212 and reverse primer from nucleotide 3319; MT-ND1, mitochondrial encoded NADH dehydrogenase 1; RPLP0, acidic ribosomal phosphoprotein P0.

| Gene | Forward 5’–3’ | Reverse 5’–3’ | Primer efficiency (%) |
| --- | --- | --- | --- |
| MTF3212/R3319 | CACCCAAGAACAGGGTTTGT | TGGCCATGGGTATGTTGTTAA | 96.3 |
| MT-ND1 | ATGGCCAACCTCCTACTCCT | CTACAACGTTGGGGCCTTT | 99.3 |
| RPLP0 | GGAATGTGGGCTTTGTGTTC | CCCAATTGTCCCCTTACCTT | 100.7 |
| ACTB | ACTCTTCCAGCCTTCCTTCC | GGCAGGACTTAGCTTCCACA | 96.8 |

**Supplementary Table S3 |** Population characteristics and perinatal factors at sampling. The numbers represent counts (percentages) for categorical and means ± standard deviation for continuous variables. mtDNA content is established by normalizing the mtDNA copy number (mean of MTF3212/R3319 and MT-ND1) to nuclear DNA copy number (mean of RPLP0 and ACTB). Cord blood mtDNA content and insulin levels are reported as median with 25^th^–75^th^ percentile. |

| Characteristics | | Study-Population (n = 179) | | | | Study-Population (n = 176) | |
| --- | --- | --- | --- | --- | --- | --- | --- |
| **Newborns** |  | |  |  | |  |  |
| Girls, n | 85 | (47%) | | 84 | | (48%) |  |
| Birthweight, grams | 3407 | ± 481.8 | | 3409 | | ±462 |  |
| European, n  160 (90.0) | 161 | (90%) | | 160 | | (91%) |  |
| Gestational age, weeks  39.1 [37-41] | 39.1 | ± 1.7 | | 39.1 | | ±1.6 |  |
| Cord blood insulin, pmol/L | 33.32 | [18.12 – 55.19] | | ─ | | ─ |  |
| Cord blood mtDNA content | ─ | ─ | | 0.96 | | [0.75 – 1.3] |  |
| **Maternal** |  |  | |  | |  |  |
| Age, years | 29.4 | ± 4.4 | | 29.3 | | ±4.3 |  |
| Pre-pregnancy BMI, kg/m^2^ | 24.1 | ± 4.3 | | 24.1 | | ±4.3 |  |
| **Education** |  |  | |  | |  |  |
| Low, n | 27 | (15%) | | 27 | | (15%) |  |
| Middle, n | 62 | (35%) | | 56 | | (32%) |  |
| High, n | 90 | (50%) | | 93 | | (53%) |  |
| **Self-reported smoking status** |  |  | |  | |  |  |
| Smoking during pregnancy, n | 26 | (14%) | | 22 | | (12%) |  |
| **Parity** |  |  | |  | |  |  |
| 1, n | 98 | (55%) | | 96 | | (55%) |  |
| ≥2, n | 81 | (45%) | | 80 | | (45%) |  |
| **Period of conception** |  |  | |  | |  |  |
| January-March | 39 | (22%) | | 42 | | (24%) |  |
| April-June | 39 | (22%) | | 36 | | (20%) |  |
| July-September | 72 | (40%) | | 69 | | (39%) |  |
| October-December | 29 | (16%) | | 29 | | (16%) |  |
|  |  | |  | |  |  |  |

## Supplementary Table S4 | The 20 highest ranked individual CpG sites according to p-value for the association with cord blood insulin in the EWAS. The number of subjects included in the two models is n = 179. β value (0–1 scale) represents the difference in methylation for a 1% change in mtDNA content relative to its reference genes. Column headers: nobs = number of observations; Gene = UCSC annotated gene; Position = chromosome and chromosomal position; Localization on CGI= UCSC gene region feature category; Localization on CGI = UCSC relation to CpG islands; β = regression coefficient; SE = standard error for regression coefficient*.*

| CpG | nobs | Gene | Position | Localization on CGI | Localization on gene | ß SE p-value | | | |
| --- | --- | --- | --- | --- | --- | --- | --- | --- | --- |
| cg25161868 | 179 | CACNA2D1 | chr7: 81579463  81579463  81579463  81579463 | Open Sea | 3'UTR | 0.022 | 0.0041 | 2.15E-07 |  |
| cg11272475 | 179 | *─* | chr22: 41460167 | Island | ─ | 0.019 | 0.0036 | 6.10E-07 |  |
| cg06434470 | 178 | DPP6 | chr7: 154429544 | Open Sea | Body | 0.015 | 0.0032 | 4.53E-06 |  |
| cg06839388 | 179 | *─* | chr14: 71152915 | Open Sea | ─ | 0.012 | 0.0027 | 1.39E-05 |  |
| cg04814784 | 179 | VHL | chr3: 10182561 | North Shore | TSS1500 | -0.240 | 0.0543 | 1.86E-05 |  |
| cg11270387 | 169 | LOC202181 | chr5: 177099251 | Island | Body | -0.004 | 0.0010 | 1.95E-05 |  |
| cg08871239 | 179 | MRPS5 | chr2: 95788279 | South Shore | TSS1500 | -0.033 | 0.0075 | 1.96E-05 |  |
| cg02274263 | 179 | SNORD115-2 | chr15: 25417786 | Open Sea | Body | 0.015 | 0.0035 | 2.43E-05 |  |
| cg21908208 | 179 | ARHGEF10L | chr1: 17865737 | North Shore | TSS1500 | 0.012 | 0.0029 | 2.58E-05 |  |
| cg24092914 | 179 | VHL | chr3: 10184877 | South Shore | Body | -0.041 | 0.0096 | 3.10E-05 |  |
| cg01148731 | 179 | MSX2 | chr5: 174156296 | North Shelf | Body | -0.036 | 0.0084 | 3.29E-05 |  |
| cg01929239 | 179 | WASH2P | chr2: 114346218 | South Shelf | Body | 0.014 | 0.0033 | 3.33E-05 |  |
| cg03542197 | 179 | SEMA3E | chr7: 83146545 | Open Sea | Body | 0.021 | 0.0049 | 3.60E-05 |  |
| cg16670245 | 178 | *─* | chr2: 196274717 | Open Sea | ─ | 0.024 | 0.0056 | 3.86E-05 |  |
| cg11221400 | 156 | TMF1 | chr3: 69101602 | Island | TSS200 | -0.017 | 0.0039 | 4.20E-05 |  |
| cg12201380 | 179 | NSMCE4A | chr10: 123717181 | Open Sea | 3'UTR | 0.043 | 0.0103 | 4.26E-05 |  |
| cg25642454 | 179 | *─* | chr12: 132909362 | North Shore | ─ | 0.015 | 0.0036 | 4.41E-05 |  |
| cg05520155 | 179 | ROBO1 | chr3: 79223592 | Open Sea | Body | 0.023 | 0.0055 | 4.71E-05 |  |
| cg23364541 | 177 | *─* | chr5: 1178961 | Open Sea | 3'UTR | -0.048 | 0.0114 | 5.18E-05 |  |
| cg08416194 | 179 | *─* | Chr5: 145236139  1.45E+08 | Open Sea | ─ | 0.0117 | 0.0028 | 5.26E-05 |  |

**Supplementary Table S5 |** The 20 highest ranked individual CpG sites according to p-value for the association with mtDNA content in the EWAS. The number of subjects included in the two models is n = 176. *β* value (0–1 scale) represents the difference in methylation for a 1% change in mtDNA content relative to its reference genes. Column headers: Gene = UCSC annotated gene; Position = chromosome and chromosomal position; Localization on CGI= UCSC gene region feature category; Localization on CGI = UCSC relation to CpG islands; β = regression coefficient; SE = standard error for regression coefficient

| CpG | nobs | Gene | Position | Localization on CGI | Localization on gene | ß | SE | p-value | |
| --- | --- | --- | --- | --- | --- | --- | --- | --- | --- |
| cg16688681 | 174 | GNA12 | chr7:2874522 | Open Sea | Body | 0.14 | 0.028 | 2.88E-06 |  |
| cg05845236 | 176 | PCDH9 | chr13:67804635 | Island | TSS200 | -0.018 | 0.004 | 3.46E-06 |  |
| cg24787924 | 176 | MIR518E | chr19:54231819 | Open Sea | TSS1500 | 0.054 | 0.011 | 4.14E-06 |  |
| cg13474520 | 174 | FKTN | chr9:108320327 | Island | TSS200 | -0.015 | 0.003 | 4.59E-06 |  |
| cg10283879 | 176 | *─* | chr3:195343007 | North Shelf | ─ | -0.045 | 0.01 | 6.92E-06 |  |
| cg27597473 | 176 | NOTCH4 | chr6:32172013 | Open Sea | Body | -0.027 | 0.006 | 7.55E-06 |  |
| cg16340918 | 176 | KIF11 | chr10:94352700 | Island | TSS200 | -0.013 | 0.003 | 9.18E-06 |  |
| cg24741703 | 176 | *─* | chr2:235459321 | Open Sea | ─ | -0.03 | 0.007 | 1.03E-05 |  |
| cg11389889 | 175 | NOP56; SNORD110;  SNORA51 | chr20:2634874 | South Shore | Body;  Body;  TSS1500 | 0.037 | 0.008 | 1.51E-05 |  |
| cg08885409 | 175 | CUEDC1 | chr17:55969571 | Open Sea | 5'UTR | 0.03 | 0.007 | 1.83E-05 |  |
| cg09334977 | 176 | *─* | chr6:170475552 | Island | ─ | 0.03 | 0.007 | 2.35E-05 |  |
| cg27633903 | 176 | MBD6 | chr12:57917206 | South Shore | 5'UTR | -0.016 | 0.004 | 2.60E-05 |  |
| ch.20.4648144R | 176 | *─* | chr20:4700144 | Open Sea | ─ | -0.03 | 0.007 | 2.78E-05 |  |
| cg24597131 | 176 | KIAA1026; | chr1:15354730 | Open Sea | Body | -0.023 | 0.005 | 2.82E-05 |  |
| cg14183927 | 176 | *─* | chr72756720 | North Shore | ─ | 0.22 | 0.052 | 3.28E-05 |  |
| cg14310768 | 175 | FBXO11; | chr2:  48133617 | North Shore | TSS1500; | -0.013 | 0.003 | 3.61E-05 |  |
| cg02606808 | 166 | MAP1B | chr5:71403502 | Island | 1stExon | -0.011 | 0.003 | 3.66E-05 |  |
| cg14583550 | 176 | FANCC | chr9:98071630 | North Shelf | 5'UTR | 0.088 | 0.021 | 3.89E-05 |  |
| cg05530592 | 176 | *─* | chr3:195355260 | North Shelf | ─ | -0.035 | 0.008 | 5.05E-05 |  |
| cg01258587 | 176 | RECK | chr9:36036735 | North Shore | TSS200 | -0.011 | 0.003 | 5.25E-05 |  |

**Supplementary Table S6 |** Pathways and gene-disease-associations found with RDAVIDWebServices exclusively for the association with cord blood insulin.

| Database | Pathway | Term | Association with cord blood insulin | | | |
| --- | --- | --- | --- | --- | --- | --- |
|  |  |  | Bonferroni-  p-value (5%) | | Count % | Fold Enrichment |
| Reactome | R-HSA-3214847 | RNA Polymerase I Promoter Opening | | 2.52E-10 | 8.96 | 10.09 |
| Reactome | R-HSA-321484 | HDMs demethylate histones | | 2.91E-05 | 4.48 | 14.33 |
| Reactome | HSA-5693571 | Nonhomologous End-Joining (NHEJ) | | 2.08E-04 | 4.48 | 11.19 |
| Reactome | R-HSA-2559580 | Oxidative Stress Induced Senescence | | 5.50E-08 | 7.46 | 9.71 |
| Reactome | R-HSA-171306 | Packaging Of Telomere Ends | | 1.14E-07 | 5.47 | 16.84 |
| Reactome | R-HSA-5693607 | Processing of DNA double-strand break ends | | 6.06E-04 | 4.98 | 8.12 |
| KEGG | hsa00860 | Porphyrin and chlorophyll metabolism | | 0.013 | 2.99 | 11.75 |
| KEGG | hsa05203 | Viral carcinogenesis | | 0.015 | 5.47 | 4.41 |
|  |  |  | |  |  |  |

**Supplementary Table S7 |** Pathways and gene-disease-associations found with RDAVIDWebServices exclusively for the association with mtDNA content.

| Database | Pathway | Term | Association with mtDNA content | | |
| --- | --- | --- | --- | --- | --- |
|  |  |  | Bonferroni-  p-value (5%) | Count % | Fold Enrichment |
| Reactome | R-HSA-1538133 | G0 and Early G1 | 0.0128 | 2.14 | 14.05 |
| Reactome | R-HSA-156711 | Polo-like kinase mediated events | 0.0024 | 1.34 | 19.64 |
|  |  |  |  |  |  |

**Supplementary Table S8 |** Pathways found with ReactomePA exclusively for the association with cord blood insulin.

| Pathway | Term | Bonferroni p-value (5%) | Count % |
| --- | --- | --- | --- |
| R-HSA-2559580 | Oxidative Stress Induced Senescence | 1.25E-10 | 12 |
| R-HSA-8939236 | RUNX1 regulates transcription of genes involved in differentiation of HSCs | 1.92E-10 | 12 |
| R-HSA-3247509 | Chromatin modifying enzymes | 3.71E-10 | 16 |
| R-HSA-3214842 | HDMs demethylate histones | 2.94E-08 | 7.2 |
| R-HSA-69473 | G2/M DNA damage checkpoint | 5.63E-08 | 8.8 |
| R-HSA-201681 | TCF dependent signaling in response to WNT | 5.63E-08 | 12.8 |
| R-HSA-8878171 | Transcriptional regulation by RUNX1 | 7.96E-08 | 12.8 |
| R-HSA-2262752 | Cellular responses to stress | 9.39E-08 | 16.8 |
| R-HSA-69481 | G2/M Checkpoints | 3.19E-07 | 10.4 |
| R-HSA-5693571 | Nonhomologous End-Joining (NHEJ) | 3.82E-07 | 7.2 |
| R-HSA-157118 | Signaling by NOTCH | 3.82E-07 | 12 |
| R-HSA-5693607 | Processing of DNA double-strand break ends | 7.47E-07 | 8 |
| R-HSA-6791312 | TP53 Regulates Transcription of Cell Cycle Genes | 6.43E-05 | 4.8 |
| R-HSA-3108232 | SUMO E3 ligases SUMOylate target proteins | 1.47E-04 | 8 |

**Supplementary Table S9 |** Reactome pathways found with ReactomePA exclusively for the association with mtDNA content.

| Pathway | Term | Bonferroni p-value (5%) | Count % |
| --- | --- | --- | --- |
| R-HSA-156711 | Polo-like kinase mediated events | 2.41E-06 | 3.39 |
| R-HSA-1538133 | G0 and Early G1 | 4.34E-05 | 3.39 |
| R-HSA-8856688 | Golgi-to-ER retrograde transport | 9.97E-05 | 6.21 |
| R-HSA-176412 | Phosphorylation of the APC/C | 9.97E-05 | 2.82 |
| R-HSA-380270 | Recruitment of mitotic centrosome proteins and complexes | 4.26E-04 | 4.52 |

**Supplementary Table S10 |** Significant differentially methylated regions with a minimal FDR value of the constituting CpGs < 0.05 as identified by the DMRcate algorithm for the association with cord blood insulin. Column headers: Gene = UCSC annotated gene; Position = chromosome and chromosomal position; Location= UCSC gene region feature category; no.cpgs = number of CpGs constituting the differentially methylated region; meanbetafc = mean ß fold change; minfdr =  minimum adjusted *p*-value from the CpGs constituting the significant region.

| Gene | Coordinates | Location | no.cpgs | meanbetafc | minfdr |
| --- | --- | --- | --- | --- | --- |
| GNAS | chr20:57426743-57428032 | promoter | 43 | 0.010 | 4.95E-09 |
| RGMA | chr15:93616424-93617168 | overlaps 5' | 14 | 0.015 | 1.20E-07 |
| ASAP3 | chr1:23763081-23763959 | overlaps 5' | 7 | -0.027 | 6.01E-05 |
| SLC6A19 | chr5:1178961-1179328 | upstream | 3 | -0.026 | 8.19E-05 |
| PARD3 | chr10:35195138-35195758 | upstream | 4 | -0.026 | 2.34E-04 |
| BRDT | chr1:92414221-92414910 | promoter | 8 | -0.030 | 4.71E-04 |
| METTL24 | chr6:110720918-110721629 | inside intron | 5 | 0.056 | 1.28E-03 |
| SYNGAP1 | chr6:33401192-33401542 | upstream | 6 | 0.012 | 2.66E-03 |
| MCF2L | chr13:113622539-113623300 | overlaps 5' | 9 | 0.012 | 2.94E-03 |
| TRAM1L1 | chr4:118006405-118006750 | overlaps 5' | 5 | 0.011 | 6.11E-03 |
| ZFAND3 | chr6:38127734-38127765 | downstream | 2 | 0.047 | 1.10E-02 |
| LOXL1 | chr15:74218529-74219307 | overlaps 5' | 10 | 0.012 | 1.73E-02 |
| CD164L2 | chr1:27709575-27709771 | overlaps exon downstream | 4 | 0.012 | 1.80E-02 |
| DDR1 | chr6:30796199-30796407 | upstream | 6 | 0.011 | 1.84E-02 |
| GRXCR2 | chr5:145236139-145236262 | downstream | 3 | -0.0081 | 1.92E-02 |
| LOC154449 | chr6:170411557-170411953 | downstream | 6 | -0.016 | 1.92E-02 |
| FAM124B | chr2:225265963-225266304 | inside exon | 3 | 0.0093 | 1.95E-02 |
| GCM2 | chr6:10884314-10884706 | promoter | 2 | -0.020 | 2.15E-02 |
| NAV1 | chr1:201618654-201619295 | inside intron | 3 | -0.019 | 2.62E-02 |
| CPT1B | chr22:51016501-51016899 | overlaps 5' | 7 | 0.029 | 3.12E-02 |

**Supplementary Table S11 |** Significant differentially methylated regions with a minimal FDR value of the constituting CpGs < 0.05 identified by the DMRcate algorithm for the association with mtDNA content. Column headers: Gene = UCSC annotated gene; Position = chromosome and chromosomal position; Location= UCSC gene region feature category; no.cpgs = number of CpGs constituting the differentially methylated region; meanbetafc = mean ß fold change; minfdr =  Minimum adjusted *p*-value from the CpGs constituting the significant region.

| Gene | Coordinates | Location | no.cpgs | meanbetafc | minfdr |
| --- | --- | --- | --- | --- | --- |
| TRIM10 | chr6:30120031-30124942 | covers exon(s) | 37 | -0.012 | 4.53E-10 |
| CYP2E1 | chr10:135340445-135343280 | overlaps 5' | 20 | 0.098 | 1.16E-07 |
| HLA-DQB2 | chr6:32729118-32731127 | covers exon(s) | 30 | -0.032 | 7.78E-06 |
| NA | chr8:685830-690059 | overlaps 5' | 15 | -0.038 | 1.75E-04 |
| CLN8 | chr8:1649522-1651197 | upstream | 8 | -0.044 | 2.89E-04 |
| MIR9-3HG | chr15:89958550-89960743 | downstream | 12 | -0.041 | 5.76E-04 |
| SLC51A | chr3:195942717-195943713 | overlaps 5' | 10 | -0.026 | 8.07E-04 |
| ARHGAP12 | chr10:32216031-32219402 | overlaps 5' | 24 | -0.013 | 8.36E-03 |
| ANKMY1 | chr2:241458886-241460664 | covers exon(s) | 9 | 0.044 | 8.84E-03 |
| H6PD | chr1:9321735-9325015 | covers exon(s) | 12 | -0.0028 | 1.06E-02 |
| EXOC3L2 | chr19:45737011-45738857 | overlaps 5' | 12 | -0.032 | 1.14E-02 |
| XXYLT1 | chr3:194797099-194797138 | inside intron | 2 | -0.056 | 1.27E-02 |
| HOXB-AS3 | chr17:46666926-46685448 | covers | 84 | -0.0099 | 1.60E-02 |
| PHGDH | chr1:120253477-120255992 | overlaps 5' | 12 | 0.0028 | 1.69E-02 |
| UNCX | chr7:1263233-1281585 | covers | 72 | -0.0067 | 1.95E-02 |
| KIAA1143 | chr3:44802549-44803638 | overlaps 5' | 19 | 0.010 | 1.95E-02 |
| TNK2 | chr3:195578011-195578280 | downstream | 6 | -0.093 | 2.14E-02 |
| DPEP1 | chr16:89686618-89687052 | overlaps 5' | 5 | -0.031 | 2.25E-02 |
| EGFR | chr7:55071456-55073797 | upstream | 9 | -0.050 | 2.80E-02 |
| SIK3 | chr11:116968354-116969870 | overlaps 5' | 9 | -0.0013 | 2.82E-02 |
| LOC286083 | chr8:1324411-1325845 | upstream | 4 | -0.035 | 3.39E-02 |
| AMZ1 | chr7:2772710-2776006 | downstream | 16 | -0.0068 | 3.81E-02 |
| ANGPT2 | chr8:6418978-6421063 | overlaps 5' | 10 | -0.067 | 4.25E-02 |
| VARS2 | chr6:30874989-30886161 | overlaps 5' | 122 | -0.0019 | 4.26E-02 |
| NKX2-3 | chr10:101279697-101285439 | upstream | 27 | -0.0020 | 4.34E-02 |
| GALNT2 | chr1:230414293-230417096 | overlaps exon upstream | 13 | 0.038 | 4.34E-02 |
| SLC45A4 | chr8:142297121-142297584 | upstream | 5 | -0.031 | 4.38E-02 |
| RPSA | chr3:39447891-39448961 | overlaps 5' | 16 | -0.0067 | 4.52E-02 |
| HCCAT5 | chr16:73205994-73207335 | downstream | 8 | -0.0011 | 4.73E-02 |
| LINC01343 | chr1:38679372-38680057 | inside intron | 2 | -0.024 | 4.76E-02 |
| LINC00906 | chr19:29217858-29218774 | upstream | 7 | 0.14 | 4.76E-02 |
| MMP10 | chr11:102638432-102638778 | downstream | 6 | 0.068 | 4.76E-02 |
| TCF19 | chr6:31122236-31127863 | overlaps 5' | 66 | -0.0066 | 4.82E-02 |
| RGMA | chr15:93956122-93959351 | upstream | 9 | -0.0063 | 4.82E-02 |

**Supplementary Table S12 |** Differentially methylated regions (DMRs) with p < 0.05 associated with cord blood insulin identified by the bumphunter algorithm. Column headers: Gene = UCSC annotated gene; Coordinates = chromosome and chromosomal position; Location= UCSC gene region feature category; no.cpgs = number of CpGs constituting the differentially methylated region; meanbetafc = mean ß fold change; FWER = Family wise error rate.

| Gene | Coordinates | Location | no.CpGs | meanbetacaf | p.value | FWER |
| --- | --- | --- | --- | --- | --- | --- |
| HOXA5 | chr7: 27182493 - 27184030 | overlaps 5' | 24 | 0.020 | 3.51E-04 | 0.21 |
| SPATC1L | chr21: 47604052 - 47605174 | overlaps 5' | 8 | -0.067 | 5.72E-04 | 0.31 |
| ZFP57 | chr6: 29648161 - 29649084 | upstream | 22 | 0.018 | 1.40E-03 | 0.64 |
| OR2L13 | chr1: 248100183 - 248101009 | overlaps 5' | 11 | 0.044 | 1.45E-03 | 0.60 |
| PM20D1 | chr1: 205818668 - 205819609 | overlaps 5' | 9 | 0.047 | 2.03E-03 | 0.73 |
| CYP2E1 | chr10: 135341528 - 135343280 | inside intron | 11 | 0.039 | 2.06E-03 | 0.72 |
| LY6G5C | chr6: 31650735 - 31651291 | covers exon(s) | 20 | 0.020 | 2.33E-03 | 0.82 |
| CPT1B | chr22: 51016501 - 51017162 | overlaps 5' | 13 | 0.026 | 2.70E-03 | 0.84 |
| MGAT1 | chr5: 180236965 - 180237008 | inside intron | 2 | 0.069 | 3.26E-03 | 0.89 |
| TRIM6 | chr11: 5616902 - 5618408 | overlaps 5' | 10 | 0.035 | 3.58E-03 | 0.91 |
| PIWIL1 | chr12: 130821607 - 130822818 | overlaps 5' | 7 | 0.045 | 3.59E-03 | 0.89 |
| HOXA5 | chr7: 27184264 - 27185512 | promoter | 17 | 0.014 | 4.87E-03 | 0.97 |
| HLA-C | chr6: 31275148 - 31276146 | upstream | 15 | 0.015 | 5.46E-03 | 0.99 |
| GNAS | chr20: 57427412 - 57427762 | promoter | 15 | 0.015 | 5.73E-03 | 0.99 |
| S100A13 | chr1: 153606541 - 153606818 | overlaps 5' | 6 | 0.041 | 6.03E-03 | 0.98 |
| MSH5 | chr6: 31712014 - 31712195 | promoter | 2 | -0.056 | 6.91E-03 | 0.99 |
| RGMA | chr15: 93616894 - 93617168 | overlaps 5' | 12 | 0.017 | 8.43E-03 | 1.00 |
| VWDE | chr7: 12443704 - 12444115 | overlaps 5' | 6 | -0.035 | 1.00E-02 | 1.00 |
| MUC4 | chr3: 195489306 - 195490309 | upstream | 9 | -0.027 | 1.00E-02 | 1.00 |
| HLA-DQB1 | chr6: 32632937 - 32633163 | promoter | 8 | 0.030 | 1.11E-02 | 1.00 |
| BRDT | chr1: 92414221 - 92414782 | promoter | 7 | -0.033 | 1.11E-02 | 1.00 |
| VEGFA | chr6: 43738006 - 43738026 | inside exon | 2 | 0.049 | 1.11E-02 | 1.00 |
| MRPL28 | chr16: 419800 - 420490 | covers exon(s) | 6 | 0.034 | 1.14E-02 | 1.00 |
| SLC25A30 | chr13: 45992561 - 45992574 | promoter | 3 | 0.042 | 1.17E-02 | 1.00 |
| RUFY1 | chr5: 178986131 - 178986906 | overlaps 5' | 9 | -0.024 | 1.32E-02 | 1.00 |
| APOB | chr2: 21266500 - 21267175 | overlaps 5' | 10 | -0.020 | 1.38E-02 | 1.00 |
| DRD4 | chr11: 637885 - 639423 | inside intron | 7 | -0.031 | 1.39E-02 | 1.00 |
| SLC9A2 | chr2: 103235539 - 103235720 | promoter | 2 | 0.045 | 1.39E-02 | 1.00 |
| LYNX1 | chr8: 143859410 - 143859990 | overlaps 5' | 8 | -0.027 | 1.52E-02 | 1.00 |
| NKX3-2 | chr4: 13546169 - 13546292 | promoter | 3 | 0.037 | 1.66E-02 | 1.00 |
| BASP1P1 | chr13: 23309774 - 23310675 | downstream | 9 | 0.022 | 1.73E-02 | 1.00 |
| FAM53A | chr4: 1579814 - 1580377 | downstream | 4 | -0.033 | 1.86E-02 | 1.00 |
| SAMD11 | chr1: 870791 - 871441 | upstream | 6 | 0.029 | 1.92E-02 | 1.00 |
| PABPC4 | chr1: 40042416 - 40042432 | inside exon | 2 | -0.039 | 2.27E-02 | 1.00 |
| RNU6ATAC | chr9: 137029881 - 137030463 | promoter | 3 | 0.034 | 2.39E-02 | 1.00 |
| AURKC | chr19: 57742112 - 57742444 | overlaps 5' | 9 | -0.018 | 2.40E-02 | 1.00 |
| DPP10 | chr2: 115419537 - 115420260 | inside intron | 7 | 0.025 | 2.43E-02 | 1.00 |
| GPR35 | chr2: 241562424 - 241562898 | promoter | 6 | 0.027 | 2.44E-02 | 1.00 |
| PLD6 | chr17: 17108846 - 17109740 | overlaps 5' | 8 | 0.021 | 2.66E-02 | 1.00 |
| RPTN | chr1: 152161237 - 152162025 | upstream | 7 | 0.024 | 2.67E-02 | 1.00 |
| NAV1 | chr1: 201618209 - 201619900 | overlaps exon downstream | 8 | -0.021 | 2.89E-02 | 1.00 |
| RNF216 | chr7: 5821227 - 5821307 | inside exon | 5 | 0.027 | 2.91E-02 | 1.00 |
| GCSAML | chr1: 247712377 - 247712591 | overlaps exon downstream | 4 | 0.029 | 2.91E-02 | 1.00 |
| TMEM18 | chr2: 731073 - 731594 | upstream | 6 | -0.025 | 2.92E-02 | 1.00 |
| ASAP3 | chr1: 23763081 - 23763959 | overlaps 5' | 7 | -0.023 | 2.98E-02 | 1.00 |
| TARP | chr7: 38350921 - 38351226 | inside intron | 5 | 0.027 | 3.00E-02 | 1.00 |
| DUSP22 | chr6: 291882 - 292522 | overlaps 5' | 6 | 0.025 | 3.12E-02 | 1.00 |
| LOXL1 | chr15: 74218418 - 74218780 | promoter | 9 | 0.014 | 3.19E-02 | 1.00 |
| ADI1 | chr2: 3486706 - 3487164 | downstream | 4 | -0.028 | 3.21E-02 | 1.00 |
| SLC23A1 | chr5: 138713954 - 138714339 | overlaps two exons | 5 | -0.026 | 3.32E-02 | 1.00 |
| HLA-E | chr6: 30419491 - 30419612 | upstream | 6 | -0.023 | 3.64E-02 | 1.00 |
| UPP1 | chr7: 48129797 - 48130197 | covers exon(s) | 6 | -0.023 | 3.73E-02 | 1.00 |
| ZNF311 | chr6: 28945182 - 28945507 | downstream | 8 | 0.018 | 3.76E-02 | 1.00 |
| ERICH1 | chr8: 599525 - 600233 | downstream | 4 | 0.026 | 3.81E-02 | 1.00 |
| HOTAIRM1 | chr7: 27137922 - 27138974 | inside intron | 7 | -0.021 | 4.00E-02 | 1.00 |
| DRD4 | chr11: 636460 - 636659 | promoter | 2 | -0.032 | 4.12E-02 | 1.00 |
| GNA12 | chr7: 2802374 - 2802697 | inside intron | 3 | -0.028 | 4.23E-02 | 1.00 |
| PCDHB7 | chr5: 140552347 - 140553150 | inside exon | 7 | 0.020 | 4.25E-02 | 1.00 |
| ZNF204P | chr6: 27343415 - 27343671 | promoter | 2 | 0.032 | 4.25E-02 | 1.00 |
| DEF8 | chr16: 90015832 - 90016551 | overlaps exon downstream | 5 | -0.024 | 4.37E-02 | 1.00 |
| TBCA | chr5: 77146796 - 77147141 | upstream | 3 | 0.028 | 4.38E-02 | 1.00 |

**Supplementary Table S13 |** Differentially methylated regions (DMRs) with p < 0. 05 associated with mtDNA content identified by the Bumphunter algorithm. Column headers: Gene = UCSC annotated gene; Coordinates = chromosome and chromosomal position; Location= UCSC gene region feature category; no.cpgs = number of CpGs constituting the differentially methylated region; meanbetafc = mean ß fold change; FWER = Family wise error rate.

| Gene | Coordinates | Location | no.CpGs | meanbetacaf | p.value | FWER |
| --- | --- | --- | --- | --- | --- | --- |
| CYP2E1 | chr10: 135341528-135343280 | inside intron | 11 | 0.17 | 6.39E-05 | 0.04 |
| PSCA | chr8: 143751447-143751801 | overlaps 5' | 5 | 0.16 | 4.41E-04 | 0.25 |
| LINC00906 | chr19: 29217858-29218774 | upstream | 7 | 0.14 | 6.80E-04 | 0.35 |
| HLA-DQB2 | chr6: 32729118-32729823 | covers exon(s) | 21 | -0.039 | 2.12E-03 | 0.80 |
| HLA-DQB1 | chr6: 32632568-32633163 | overlaps 5' | 11 | -0.067 | 3.31E-03 | 0.89 |
| TBCD | chr17: 80889693-80890438 | inside intron | 4 | -0.11 | 3.52E-03 | 0.90 |
| ZXDC | chr3: 126194903-126194992 | promoter | 5 | -0.10 | 3.88E-03 | 0.93 |
| RUFY1 | chr5: 178986131-178986906 | overlaps 5' | 9 | -0.077 | 4.09E-03 | 0.95 |
| PSORS1C3 | chr6: 31148332-31148748 | upstream | 14 | 0.035 | 5.81E-03 | 0.99 |
| OR2L13 | chr1: 248100183-248101009 | overlaps 5' | 11 | -0.052 | 6.36E-03 | 0.99 |
| MUC4 | chr3: 195488725-195490309 | upstream | 11 | -0.051 | 6.68E-03 | 0.99 |
| CPT1B | chr22: 51016501-51017162 | overlaps 5' | 13 | 0.037 | 6.83E-03 | 0.99 |
| LAIR2 | chr19: 55013821-55014066 | overlaps 5' | 5 | -0.085 | 7.29E-03 | 0.99 |
| NBR2 | chr17: 41278135-41278906 | inside intron | 13 | 0.033 | 7.80E-03 | 1.00 |
| ACY3 | chr11: 67417958-67418405 | overlaps 5' | 12 | 0.036 | 8.36E-03 | 1.00 |
| SDHAF1 | chr19: 36486125-36486149 | inside exon | 2 | -0.11 | 9.26E-03 | 1.00 |
| DRD4 | chr11: 636460-636659 | promoter | 2 | -0.10 | 1.07E-02 | 1.00 |
| ALKBH7 | chr19: 6373461-6373627 | inside intron | 2 | 0.10 | 1.09E-02 | 1.00 |
| PIWIL1 | chr12: 130821607-130822818 | overlaps 5' | 7 | 0.068 | 1.12E-02 | 1.00 |
| ZFP57 | chr6: 29648525-29649084 | upstream | 10 | -0.046 | 1.15E-02 | 1.00 |
| HLA-DRB6 | chr6: 32551749-32552246 | overlaps 5' | 10 | -0.045 | 1.22E-02 | 1.00 |
| ZNF556 | chr19: 2866619-2866899 | promoter | 2 | -0.098 | 1.23E-02 | 1.00 |
| LOC643802 | chr16: 53406901-53407808 | promoter | 9 | -0.052 | 1.26E-02 | 1 |
| KRTCAP3 | chr2: 27665017-27665543 | overlaps 5' | 9 | 0.051 | 1.28E-02 | 1 |
| MIR9-3HG | chr15: 89959984-89960743 | downstream | 7 | -0.063 | 1.44E-02 | 1 |
| AURKC | chr19: 57741988-57742444 | overlaps 5' | 10 | 0.040 | 1.51E-02 | 1 |
| CCKBR | chr11: 6291549-6292896 | covers | 10 | 0.039 | 1.55E-02 | 1 |
| HNRNPM | chr19: 8509335-8509471 | promoter | 3 | -0.081 | 1.56E-02 | 1 |
| ANKMY1 | chr2: 241458886-241460002 | covers exon(s) | 8 | 0.055 | 1.60E-02 | 1 |
| DUSP22 | chr6: 291687-293285 | overlaps 5' | 10 | -0.038 | 1.62E-02 | 1 |
| COLEC11 | chr2: 3642400-3642967 | overlaps 5' | 9 | -0.045 | 1.75E-02 | 1 |
| GABBR1 | chr6: 29599012-29599390 | covers exon(s) | 10 | -0.036 | 1.77E-02 | 1 |
| SPATC1L | chr21: 47604052-47605174 | overlaps 5' | 8 | 0.052 | 1.81E-02 | 1 |
| PRDM9 | chr5: 23507030-23507656 | promoter | 8 | 0.051 | 1.92E-02 | 1 |
| ZNF497 | chr19: 58874030-58874233 | overlaps 5' | 2 | -0.085 | 1.95E-02 | 1 |
| AMZ1 | chr7: 2764209-2764246 | downstream | 2 | -0.083 | 2.06E-02 | 1 |
| TCP11L2 | chr12: 106696891-106697297 | overlaps 5' | 3 | 0.073 | 2.20E-02 | 1 |
| BASP1P1 | chr13: 23309774-23310675 | downstream | 9 | 0.039 | 2.22E-02 | 1 |
| DRD4 | chr11: 637885-639423 | inside intron | 7 | -0.053 | 2.39E-02 | 1 |
| GALNT2 | chr1: 230415185-230415668 | inside exon | 6 | 0.057 | 2.40E-02 | 1 |
| MTG1 | chr10: 135202522-135203200 | upstream | 7 | -0.052 | 2.53E-02 | 1 |
| ALOX12 | chr17: 6899207-6899577 | overlaps 5' | 9 | 0.035 | 2.71E-02 | 1 |
| KCNJ1 | chr11: 128693961-128694679 | downstream | 7 | -0.050 | 2.75E-02 | 1 |
| DNAJC17 | chr15: 41100233-41100308 | promoter | 2 | 0.076 | 2.76E-02 | 1 |
| LINC01101 | chr2: 121223534-121224009 | overlaps 5' | 7 | -0.050 | 2.83E-02 | 1 |
| GTPBP3 | chr19: 17448797-17449202 | covers exon(s) | 4 | 0.060 | 2.99E-02 | 1 |
| FZD6 | chr8: 104309910-104310606 | promoter | 3 | -0.066 | 3.02E-02 | 1 |
| HLA-E | chr6: 30420981-30421275 | upstream | 7 | 0.048 | 3.13E-02 | 1 |
| HOXA5 | chr7: 27183369-27183701 | promoter | 9 | 0.028 | 3.19E-02 | 1 |
| CACNA2D4 | chr12: 2018050-2018144 | covers exon(s) | 2 | -0.073 | 3.20E-02 | 1 |
| TMEM204 | chr16: 1583810-1584516 | inside exon | 8 | -0.040 | 3.24E-02 | 1 |
| KLHDC7B | chr22: 50985681-50986813 | overlaps 5' | 6 | -0.050 | 3.49E-02 | 1 |
| GP6 | chr19: 55549414-55549842 | overlaps 5' | 7 | -0.045 | 3.62E-02 | 1 |
| CFAP161 | chr15: 81426434-81426820 | overlaps 5' | 8 | -0.037 | 3.80E-02 | 1 |
| SAMD11 | chr1: 870791-871546 | upstream | 7 | 0.044 | 3.81E-02 | 1 |
| POU2AF1 | chr11: 111250093-111250338 | overlaps 5' | 6 | -0.048 | 3.81E-02 | 1 |
| CREBBP | chr16: 3988694-3988869 | upstream | 3 | -0.062 | 3.88E-02 | 1 |
| TAGLN | chr11: 117069780-117070046 | overlaps 5' | 6 | 0.048 | 3.90E-02 | 1 |
| GCSAML | chr1: 247712512-247712591 | overlaps exon downstream | 2 | 0.068 | 3.90E-02 | 1 |
| EXOC3L2 | chr19: 45737603-45738115 | promoter | 7 | -0.043 | 4.12E-02 | 1 |
| FILIP1 | chr6: 76203225-76203675 | overlaps 5' | 7 | -0.042 | 4.37E-02 | 1 |
| KIAA1143 | chr3: 44802549-44802863 | inside intron | 6 | 0.046 | 4.38E-02 | 1 |
| VTA1 | chr6: 142468350-142468432 | overlaps 5' | 5 | 0.049 | 4.58E-02 | 1 |
| PGAM2 | chr7: 44104584-44105434 | overlaps 5' | 6 | -0.045 | 4.71E-02 | 1 |
| JMJD4 | chr1: 227922838-227923227 | overlaps 5' | 3 | -0.057 | 4.85E-02 | 1 |
| IGF2BP1 | chr17: 47091339-47092272 | inside intron | 7 | 0.039 | 4.87E-02 | 1 |
| ZNF678 | chr1: 227746111-227747468 | upstream | 7 | 0.039 | 4.87E-02 | 1 |
| TESC | chr12: 117484926-117484975 | inside intron | 3 | 0.057 | 4.90E-02 | 1 |
| DAZAP1 | chr19: 1407376-1407468 | promoter | 2 | 0.064 | 4.93E-02 | 1 |

1. **Supplementary text |** Difference in program algorithms

DMRcate

For DMRcate the matrix of M-values is annotated first with information about the genomic position of the probes using the ilmn12.hg19 annotation (75) and UCSC Genome Browser. In the process of finding DMRs itself DMRcate is agnostic to all annotations except for spatial ones like chromosomal coordinates. Then, like in the analysis of individual CpG sites, a limma linear model with empirical Bayes adjustment is fitted for each individual CpG site. Subsequently the individual CpG sites are combined by the 'dmrcate' function and modeled by Gaussian Kernel smoothing. Notably for this procedure, DMRcate uses unsigned weights (limma’s t^2^s) to pass to the kernel estimator were the estimates are calculated according to the formula: $K_{ij}=exp \left( \frac{{-[x_{i}-x_{j}]}^{2}}{2\sigma^{2}} \right),$ with the Gaussian kernel weights represented by *K*_ij_ for the *F* statistics, *Y*_i_ at the locations *x*_i_, and the kernel scale factor *σ* proportional to the bandwidth *λ*. By this, two estimates, one weighted and one not, are derived. For each chromosome these two smoothened estimates are subsequently compared via a Satterthwaite approximation and a significance test is conducted. Probes significant after FDR correction and within a distance of λ nucleotides to each other are then grouped into a region.

Bumphunter:

Bumphunter also uses smoothed methylation values to discover DMRs but in contrast to DMRcate the bumphunter algorithm first defines clusters of probes and tests for significance afterwards. Another difference with DMRcate is that bumphunter works under the assumption that methylation changes of CpGs within a given region must always be in the same direction. Consequently, signed weights are passed on to the smoothing process, which can cause a loss of biologically significant results when positive and negative signs cancel each other out. Bumphunter first computes a t-statistic for each genomic location by applying a regression model for the logit-transformed methylation measurements against the variable of interest. The estimated slope $\hat{ß}$(_tj_) is retained and loess smoothing is applied to create clusters. A designated candidate region is subsequently formed by clusters of nearby probes for which all the t-statistics exceeded a predefined cut-off threshold chosen by the program with a default of 0.99. Finally, permutation or bootstrapping procedures that construct null distributions for each candidate region are employed.
